# Plasmidome Interchange between *Clostridium botulinum, Clostridium novyi* and *Clostridium haemolyticum* Converts Strains of Independent Lineages into Distinctly Different Pathogens

**DOI:** 10.1371/journal.pone.0107777

**Published:** 2014-09-25

**Authors:** Hanna Skarin, Bo Segerman

**Affiliations:** 1 Department of Bacteriology, National Veterinary Institute (SVA), Uppsala, Sweden; 2 Department of Biomedical Sciences and Veterinary Public Health, Swedish University of Agricultural Sciences (SLU), Uppsala, Sweden; The University of Melbourne, Australia

## Abstract

*Clostridium botulinum* (group III), *Clostridium novyi* and *Clostridium haemolyticum* are well-known pathogens causing animal botulism, gas gangrene/black disease, and bacillary hemoglobinuria, respectively. A close genetic relationship exists between the species, which has resulted in the collective term *C. novyi sensu lato*. The pathogenic traits in these species, e.g., the botulinum neurotoxin and the novyi alpha toxin, are mainly linked to a large plasmidome consisting of plasmids and circular prophages. The plasmidome of *C. novyi sensu lato* has so far been poorly characterized. In this study we explored the genomic relationship of a wide range of strains of *C. novyi sensu lato* with a special focus on the dynamics of the plasmidome. Twenty-four genomes were sequenced from strains selected to represent as much as possible the genetic diversity in *C. novyi sensu lato.* Sixty-one plasmids were identified in these genomes and 28 of them were completed. The genomic comparisons revealed four separate lineages, which did not strictly correlate with the species designations. The plasmids were categorized into 13 different plasmid groups on the basis of their similarity and conservation of plasmid replication or partitioning genes. The plasmid groups, lineages and species were to a large extent entwined because plasmids and toxin genes had moved across the lineage boundaries. This dynamic process appears to be primarily driven by phages. We here present a comprehensive characterization of the complex species group *C. novyi sensu lato,* explaining the intermixed genetic properties. This study also provides examples how the reorganization of the botulinum toxin and the novyi alpha toxin genes within the plasmidome has affected the pathogenesis of the strains.

## Introduction

The three anaerobic Gram-positive spore-forming bacterial species, *Clostridium botulinum*, *Clostridium novyi* and *Clostridium haemolyticum,* are pathogens that affect animals and humans worldwide. They are widely spread in the environment, with reservoirs both in soil and water sediments [1], [2], [3]. Extrachromosomal elements play a major role in the diseases caused by these species: animal botulism (*C. botulinum* group III), gas gangrene (*C. novyi*), black disease (*C. novyi*) and bacillary hemoglobinuria (*C. haemolyticum*). Although causing different diseases, they are clearly genetically related. Yet until now, the relationship between these species on a genomic level has only fragmentally been described [4], [5], [6].

*C. botulinum* is recognized by its capacity to produce botulinum neurotoxins (BoNTs) of serotypes A–G. These cause botulism by blocking acetylcholine release in peripheral nerves, thereby resulting in paralysis. The species is usually divided into four distantly related groups on the basis of genetic and physiological similarities [7]. This study concerns *C. botulinum* group III, which is associated with animal botulism. Strains belonging to this group carry *bont* genes of types C, and D or chimerical variants referred to as types C/D and D/C. The *bont* genes in *C. botulinum* group III are carried by bacteriophages, which propagate as large unstable plasmids that are frequently lost during isolation and *in vitro* cultivation [8]. *C. botulinum* group III strains produce several additional toxins, whose role in pathogenesis is less understood [6]. *C. novyi* strains are also classified into different types: A, B and C, on the basis of the toxins they produce. Both *C. novyi* type A and type B produce a lethal alpha-toxin, which is encoded by a bacteriophage [9]. *C. novyi* type A and B strains also produce phospholipases, which differ serologically from each other and are designated gamma- and beta toxin, respectively [10]. The gamma toxin is a non-lethal phospholipase, whereas the beta toxin of *C. novyi* type B lyses hepatocytes and erythrocytes and damages capillary endothelium [11]. *C. novyi* type C produces only gamma toxin and is not associated with any disease. *C. novyi* type A, and to some extent type B, can cause tissue infections (gas gangrene) in both humans and animals [10], [12]. In addition, type B causes infectious necrotic hepatitis, so-called black disease, primarily in sheep but also in other ruminants and occasionally in pigs and horses [10], [13], [14]. The only virulence factor known to be produced by *C. haemolyticum* is the beta toxin, and it is serologically identical to that of *C. novyi* type B [10]. *C. haemolyticum* causes a rapidly fatal disease known as bacillary hemoglobinuria, which primarily affects cattle but also other ruminants [15], [16], [17].

Before the era of next-generation sequencing, descriptions of genetic relatedness between the three species were based on 16S rRNA similarity and DNA-DNA hybridizations [4], [5]. When the first genome of a *C. botulinum* group III organism was completed, comparisons were made to the existing chromosomal sequence of a *C. novyi* type A strain. The chromosomes were discovered to be so similar that the collective “genospecies” name *C. novyi sensu lato* was proposed [6]. Before the present study, there were five genome sequences of *C. botulinum* group III isolates and one of *C. novyi* type A available in public databases, together with a handful of plasmids or bacteriophages from *C. botulinum* group III strains [6], [18], [19], [20], [21]. Remarkably, there were no plasmids or bacteriophage sequences from *C. novyi* or *C. haemolyticum*. Here we present a study on genomic relationships and a comprehensive characterization of the plasmids and phages and their movements within the *C. novyi sensu lato* group.

## Results

### Four phylogenomic lineages could be identified in *C. novyi sensu lato*, each represented by strains with different characteristics and pathogenic capacity

**Figure 1:**
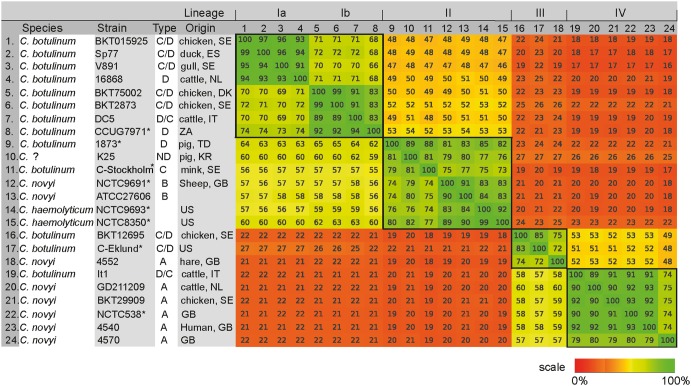
Average whole genome similarity of *C. novyi sensu lato* genomes. A similarity matrix based on normalized average BLASTN sores for fragmented comparisons covering the whole genomes (200 bp fragment size) illustrated by a heat-plot. Four lineages were identified (I–IV) and are framed with black squares. The geographical origins of the strains are listed with two-digit country codes. Strains isolated more than 50 years ago are marked with a star.

The strains in this study were selected on the basis of pulsed-field gel electrophoresis (PFGE) subtyping data (data not shown) to give the widest possible representation of the genetic diversity within *C. novyi sensu lato,* different geographic origins, and animal hosts. Whole genome sequence assemblies were produced and compared by calculating pairwise average BLASTN score similarities over the whole genomes. Four lineages could be defined from the 24 strains compared (Figure 1). The branch represented by lineages I and II was relatively diverged from that represented by linages III and IV. Designated species, toxin type, geographical origin, and the host from which the strains were isolated, were in most cases variable within the lineages (Figure 1). The sequencing statistics and the quality assessments of the genome assemblies are summarized in Table S1.

Lineage I consisted solely of *C. botulinum* group III strains. Two separable subgroups, Ia and Ib, of closely related strains could be distinguished. All strains in lineage I had been isolated during the last decade, except the type D strain in subgroup Ib, which dated back to 1929. Strains represented toxin types D, C/D, and D/C and a wide geographic distribution with origins including Sweden, Holland and Spain, Denmark, Italy and South Africa. The C/D strains were from avian sources whereas the D and D/C strains were bovine (in one case, the source was unknown). The genomes in lineage I, and especially lineage Ia, had a higher frequency of insertion sequence (IS) elements compared to the other lineages. Of the five different IS elements found in a high copy-number in lineage Ia [6], no more than two were found in any of the strains in lineage II–IV (Table S2).

Lineage II was represented by strains from all three species: *C. botulinum*, *C. haemolyticum* and *C. novyi*. Most strains were isolated more than 50 years ago, except for strain k25, which was isolated in South Korea in 2012. For those strains where the isolation source was known, the origins were mink, pig and sheep. The geographic distribution was broad, with strains from Chad, Korea, Sweden, Great Britain and the United States.

Lineage III was represented by strains of *C. botulinum* and *C. novyi*. These two *C. botulinum* strains were both of toxin type C/D, and were isolated in the United States in the middle of the 20^th^ century and in Sweden 2010, respectively. The *C. novyi* strain was classified as type A and had been isolated from hare in Great Britain in 2000. Although the Swedish *C. botulinum* strain and the *C. novyi* strain had lost their toxin-encoding phages when sequenced, they were previously confirmed toxin positive by PCR [22].

Lineage IV was represented by *C. novyi* strains and one *C. botulinum* strain. The *C. novyi* strains were type A, originated from Italy, the Netherlands, Sweden or Great Britain, and had been isolated from cattle, chicken or human samples. The *C. botulinum* strain was isolated from cattle diagnosed with botulism of type D/C, but the strain had subsequently lost the toxin gene. All the lineage IV strains originated from the last decade, except for one *C. novyi* strain, which was from 1920.

### *C. novyi sensu lato* has a large plasmidome, which could be divided into 13 plasmid groups

Within the 24 genomes, 61 plasmids were identified. Nine of those were previously published completed plasmids, 28 we processed during this study into completed, annotated circular plasmids (Figure S1), and the remaining 24 plasmids were represented by draft plasmid continuous sequences (contigs). Plasmids were identified by a combination of PFGE analysis (Figure S2), comparisons of sequence contigs obtained before and after plasmid curation, and by detailed characterization of individual WGS contigs. The plasmids ranged from 12 kb to 204 kb in size. The genomic properties of these 61 plasmids are summarized in Table S3. Strains identified as *C. botulinum* group III, *C. novyi* type B or *C. haemolyticum* typically contained between three and five plasmids each, whereas strains of *C. novyi* type A contained only one plasmid, *i.e.,* the alpha-toxin encoding bacteriophage.

**Figure 2:**
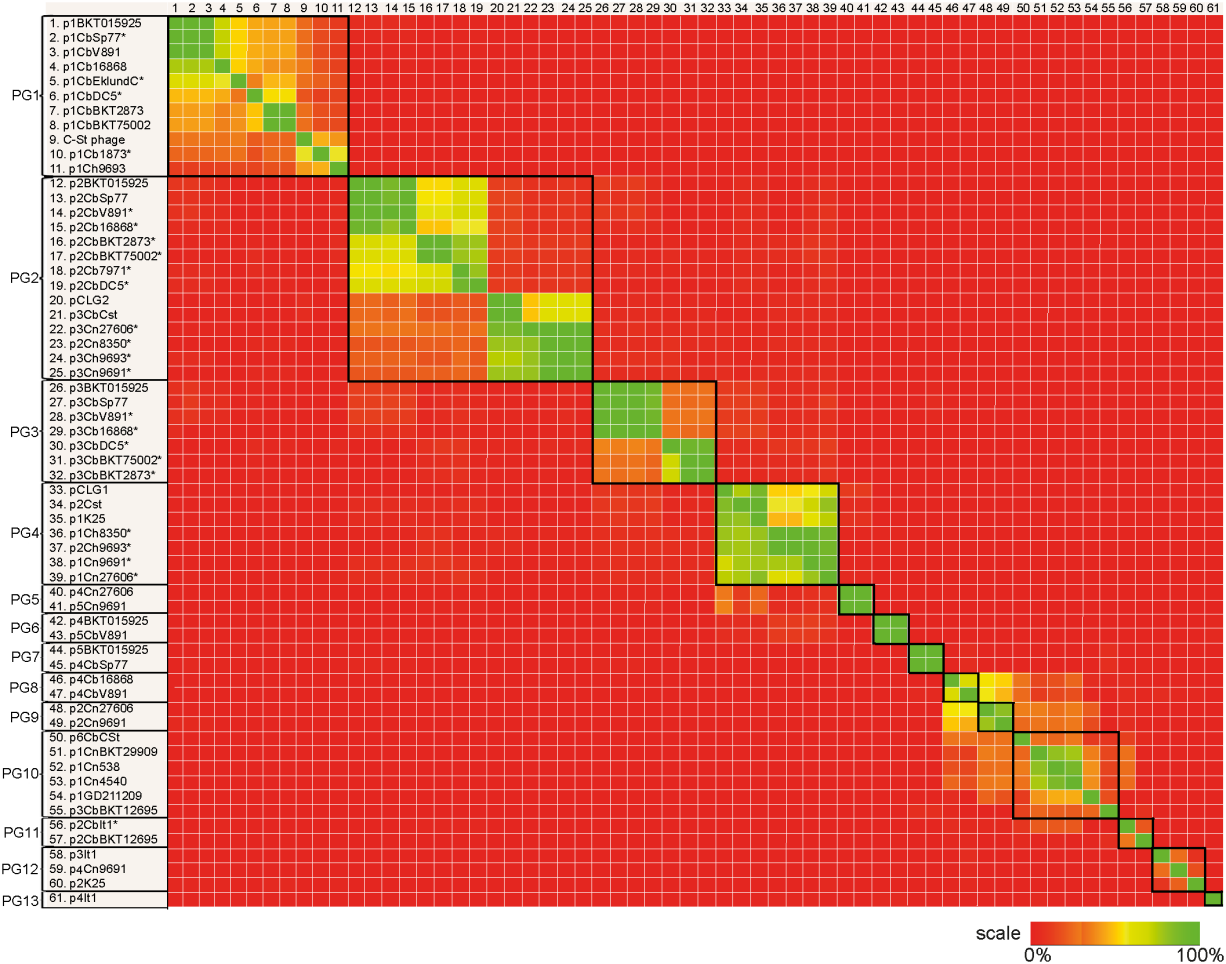
Comparative analysis of *C. novyi sensu lato* plasmids. A heat-plot of the relative amount of shared genetic material between plasmids (40% normalized BLASTN score threshold). Thirteen plasmid groups (PG1–PG13), framed by black squares, were identified from these results and from analysis of plasmid replication and partitioning genes. Some of the plasmids in lineage I strains (PG1–PG3) shared several IS elements, and this resulted in an apparent increase in the background level of shared genetic content between otherwise dissimilar plasmids within this lineage. Uncompleted plasmids are marked with a star.

The plasmids showed a large variability in size and gene content. In order to identify groups of related plasmids, the pairwise relative amount of conserved genetic material was determined and visualized as a heat-plot (Figure 2). The full heat-plot, including the values from which the color codes were determined, is represented as Figure S3. The overall gene conservation, in combination with the conservation of specific plasmid replication and partitioning genes, were considered for categorizing the plasmids into 13 groups (Figure 2, Table 1, Table S5 and description below). Six of these plasmid groups (PG5 and PG8–PG13) have never been genetically described before. Phage structural genes were commonly found in many of the plasmids, but PG2–PG5 and PG13 contained no phage related genes and were therefore considered to be regular plasmids.

**Table 1:** Representation of plasmid groups and the plasmid distribution in analyzed strains.

Plasmid group				1	2	3	4	5	6	7	8	9	10	11	12	13
L	Sp	Type/Strain	No													
Ia	B	CD/BKT015925	5	x	x	x			x	x						
	B	CD/Sp77	4	x	x	x			x							
	B	CD/V891	5	x	x	x				x	x					
	B	D/16868	3	x	x	x					x					
Ib	B	CD/BKT75002	3	x	x	x										
	B	CD/BKT2873	3	x	x	x										
	B	DC/DC5	3	x	x	x										
	B	D/CCUG 7971	2	x*	x											
II	B	D/1873	3	x	x		x									
	?	K25	2				x								x	
	B	C/C-Stockholm	4	x	x		x						x			
	N	B/NCTC 9691	5		x		x	x				x			x	
	N	B/ATCC 27606	4		x		x	x				x				
	H	NCTC 9693	3	x	x		x									
	H	NCTC 8350	2		x		x									
III	B	CD/BKT12695	3	x*									x	x		
	B	CD/C-Eklund	1	x												
	N	A/4552	1										x*			
IV	B	DC/It1	4	x*										x	x	x
	N	A/GD211209	1										x			
	N	A/BKT29909	1										x			
	N	A/NCT C538	1										x			
	N	A/4540	1										x			
	N	A/4570	1										x*			

The table headings represent Lineage (L), Species (Sp), Type and Strain name (Type/Strain) and number of plasmids predicted in each strain (No). The species are represented by B (*Clostridium botulinum*), N (*Clostridium novyi*) or H (*Clostridium haemolyticum*).

*The sequenced strain lacked the plasmid but was previously confirmed positive for it.

#### PG1

All plasmids had similar sizes (170–200 kb) and contained many phage related genes. All annotated plasmids contained a conserved *ftsZ* gene coding for a potential segrosome component (Table S5), and corresponding DNA sequence was found in also remaining uncompleted PG1 plasmids (>97 nucleotide sequence identity and 100% coverage). FtsZ proteins have previously been implicated in segrosome based partitioning of large plasmids in *Bacillus* spp. [23]. The plasmids in this group also encoded proteins with putative DNA helicase, DNA primase, DNA polymerase III and DNA gyrase functions (Table S5), but the sequences were not highly conserved within the group (down to less than 30% identity on protein level).

#### PG2

The plasmids from lineage I had similar sizes (80–100 kb), whereas the plasmids from lineage II were around half these sizes. Both annotated plasmids contained a conserved *ftsZ* gene (Table S5), and corresponding DNA sequence was found also in the uncompleted PG2 plasmids (>99 nucleotide sequence identity and 100% coverage). This gene was related to the *ftsZ* gene found in PG1 (∼30% amino acid sequence identity). Further, a conserved DnaD domain containing gene, possibly involved in replication initiation, was present (Table S5). The putative DNA helicase, DNA primase, DNA polymerase III and DNA gyrase encoding genes found in PG1 were not present in PG2.

#### PG3

All plasmids belonged to lineage I and were approximately 80 kb. There was a phylogenetic distinct difference between the plasmids from lineage Ia and plasmids from lineage Ib, which was more pronounced than corresponding difference seen in PG1 and PG2. The low or nonexistent sequence similarity to the other plasmids groups was the criterion for creating this plasmid group. Replication related genes could not be found, but a conserved putative prevent-host-death protein was present (Table S5).

#### PG4

These plasmids were only found in lineage II and had a broad size range, between approximately 60 and 100 kb. The two completed and annotated plasmids in PG4 (p1K25 and pCLG1) contained one conserved plasmid replication initiation gene (Table S5), which was conserved in also remaining uncompleted plasmids in PG4 (>97% nucleotide sequence identity and 100% coverage) and was thus considered a marker for this group.

#### PG5

The two 30 kb plasmids (p5Cn9691 and p4Cn27606) belonged to *C. novyi* type B strains in lineage II. They shared more than 99% of their genetic content. Two conserved genes encoding proteins involved in plasmid replication (Table S5), which were not found in any of the other plasmid groups, were considered markers for this group.

#### PG6

The two 40 kb plasmids in this group, p4BKT015925 and p5CbV891, both belonged to strains of lineage Ia, and they shared more than 96% of their genetic content. They were identified as prophages due to the high number of phage structural genes. This plasmid group contained three genes (Table S5) encoding proteins involved in plasmid replication and partitioning with no or low sequence similarity to genes with similar functions in the other plasmid groups.

#### PG7

The two 12 kb plasmids (p5BKT015925 and p4CbSp77) belonged to *C. botulinum* strains of lineage Ia and shared more than 99% of their genetic content with each other. They contained many phage related genes. No genes coding for plasmid replication or segregation were annotated in these plasmids and therefore no plasmid group marker was identified. The high sequence similarity between the two plasmids and the low or nonexistent similarity to the other plasmids was the criterion for creating this plasmid group.

The division of plasmids into PG8–PG11 was made on the basis on the overall conservation of genes surrounding their interchangeable phage structural modules, in addition to identified plasmid group markers.

#### PG8

The two 58 kb plasmids (p4CbV891 and p4Cb16868) belonged to *C. botulinum* strains of lineage Ia. One gene encoding a protein predicted to be involved in plasmid partitioning was conserved in PG8 (Table S5), and showed no or low sequence similarity to genes in other plasmid groups.

#### PG9

The two 60 kb and 57 kb plasmids (p2Cn9691 and p2Cn27606) belonged to *C. novyi* type B strains of lineage II. Two genes encoding proteins predicted to be involved in plasmid replication were conserved in PG9 (Table S5), and showed no or low similarity to genes in other plasmid groups.

#### PG10

This plasmid group included four *C. novyi* type A plasmids (p1CnBKT29909, p1CnGD211209, p1Cn538, p1Cn4540) of approximately 60 kb, and one 55 kb and one 38 kb plasmid found in *C. botulinum* strains in lineage II and III, respectively (p6CSt and p3CbBKT12695). One gene encoding a protein predicted to be involved in plasmid segregation was conserved in PG10 (Table S5), and showed no or low similarity to genes in other plasmid groups. The nucleotide sequence spanning over this region in p6CSt contained several stop codons, but the complete sequence was covered and was 90% similar to the corresponding gene in p1CnBKT29909.

#### PG11

The two 52 kb and ∼87 kb plasmids (p2CbBKT12695 and p2CbIt1) belonged to *C. botulinum* strains of lineage III and IV, respectively. No genes coding for proteins involved in plasmid replication or segregation were annotated in PG11 and therefore no plasmid group marker was identified. These plasmids were categorized into the same plasmid group on the basis of the conserved sequences surrounding the differing phage structural modules.

#### PG12

Two of the plasmids: p2K25 and p4Cn9691 (43 and 51 kb, respectively, were found in lineage II and one, p3CbIt1 (51 kb), was found in lineage IV. All three were identified as prophages due to their high phage structural gene content. The three plasmids all contained a gene encoding a plasmid segregation protein ParM (Table S5). Plasmid p4Cn9691 shared approximately 18% of its genetic content with both the other plasmids, whereas they did not share other genes than the gene marker. Therefore, all three plasmids were assumed to contain different structural phage modules, but the same DNA segregation module. A similar (72% amino acid identity) *parM* gene was also found in one of the PG8 plasmids (Table S5). The sharing of the same DNA segregation module, in combination with low or nonexistent sequence similarity to the other plasmids groups, was the criterion for creating this plasmid group.

#### PG13

The single plasmid (p4CbIt1) in this group was 13 kb in size and belonged to a *C. botulinum* strain in lineage IV. It contained one gene coding for a replication protein (Table S5) with no or low sequence similarity to genes with similar functions in the other plasmid groups.

The plasmid groups, lineages, and species, were to a large extent, entwined. Five plasmid groups (PG1, PG2, PG10, PG11 and PG12) were represented by more than one lineage and five plasmid groups (PG1, PG2, PG4, PG10, PG12) by more than one species (Table 1). Plasmid groups PG1, PG10, and PG12 were found both in the lineage I–II and III–IV branches, whereas PG2-PG9 were found only in the lineage I–II branch and plasmid groups PG11 and PG13 only in the lineage III–IV branch. Although the *C. novyi* strains of lineage III and IV contained only a single plasmid (PG10) each, the *C. botulinum* group III strains from the same lineages contained several plasmids, including the ones unique for this branch (PG11 and PG13).

Only two putative conjugation related genes were identified in the annotated plasmids of *C. novyi sensu lato* (p3CbIt1, Z963-p0074 and p2CbBKT12695, Z962-p0079). The PG1 and PG2 plasmid sequences derived from lineages I and II, when analyzed separately, could be divided into the same phylogenetic groups as their corresponding whole genomes, suggesting that propagation of these plasmids, to a large extent, remains within their lineages. The same conclusion could be drawn for the plasmid groups that were only connected with one lineage: PG3-PG9 and PG13. The plasmid groups that contained plasmids harboring phage structural genes and therefore likely were to be circularized prophages (PG1, PG6–12) were mainly those that were lost in the plasmid curation experiments and consequently less stable in the population than the regular plasmids (Table S4). As exemplified below, the interactions between lineages have mainly been through phage and horizontal gene movements.

### The BoNT-encoding plasmid group (PG1) consists of large unstable prophages, existing in all lineages but most common in lineages I and II

The plasmids in PG1 have previously been shown experimentally to be prophages [24]. It is well known that PG1 plasmids are unstable in many strains and can be readily lost during isolation or *in vitro* propagation. When possible, we selected isolates that had retained the plasmid, but in order to get representative sequences from all lineages, we had to select a few strains that had lost their PG1 plasmid. The PG1 plasmids existed in all lineages, but the only available *C. botulinum* strain in lineage IV had lost its plasmid when sequenced. In lineages III and IV, two out of three PG1 plasmids had been lost during cultivation. The only PG1 plasmid from these lineages for which we could obtain a sequence (p1CbCEklund), has probably been acquired horizontally from lineage I, given its similarity to the PG1 sequences of lineage I (Figure 2). This means that all plasmids we have sequenced so far encoding a chimeric botulinum toxin (C/D or D/C) originate from lineage I.

### Plasmids in PG1 contain typically, but not always, the bont gene cluster

**Figure 3:**
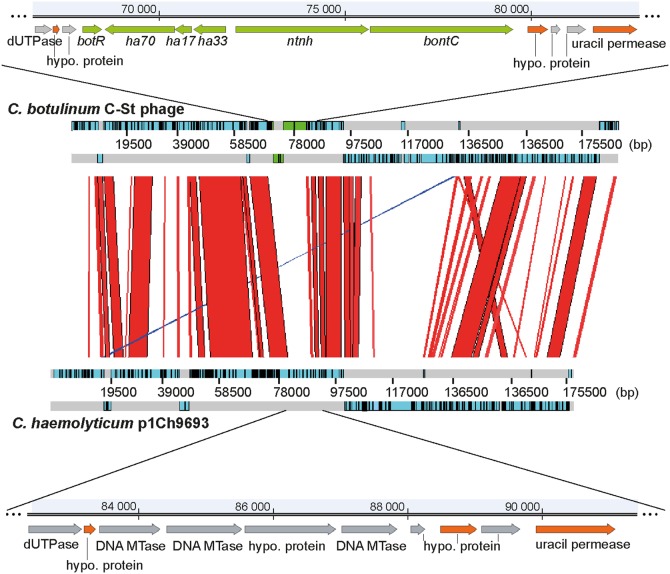
Sequence comparison between the C-St BoNT-encoding phage and the p1Ch9693 phage from a *C. haemolyticum* strain. Artemis Comparison Tool (ACT) plot of an alignment between the C-St BoNT-encoding phage from *C. botulinum* strain C-Stockholm and p1Ch9693 from *C. haemolyticum* strain NCTC 9693. Regions of similarity are indicated in red (same direction) or blue (opposite direction). Genes in the *bont* cluster are green, genes homologous between the two replicons are orange, and remaining genes are gray.

All the PG1 plasmids from *C. botulinum* strains contained a gene encoding the C3 toxin, and the *bont* gene cluster, which is a prerequisite for a strain to be classified as *C. botulinum*. We also identified a plasmid in *C. haemolyticum* strain NCTC 9693, which lacked the *bont* gene cluster but was otherwise similar to the PG1 plasmids; it was most closely related to the PG1 plasmid from *C. botulinum* strain C-Stockholm (Figure 3). The *bont* gene cluster (*bot-R*, *ha-70*, *ha-17*, *ha-33*, *ntnh* and *bont*) was found at approximately the same position, i.e., a short distance downstream a conserved gene coding for a UTPase (>97% amino acid identity). In one strain, 16868 (lineage Ia), a complete ISCbo1 transposon was found downstream the *bont* gene cluster (Figure S4). The nucleotide sequences of the *bont* genes within each serotype were well conserved (>99% nucleotide identity) and the few single nucleotide polymorphisms (SNP) reflected lineage identity (data not shown). Comparing all the *C. botulinum* group III BoNT serotypes, the N-terminal domain of the heavy chain was the most conserved part, followed by the light chain. The C-terminal domain of the heavy chain was the least conserved part.

### Chimeric forms of the bont gene arose by recombination in the heavy chain

**Figure 4:**
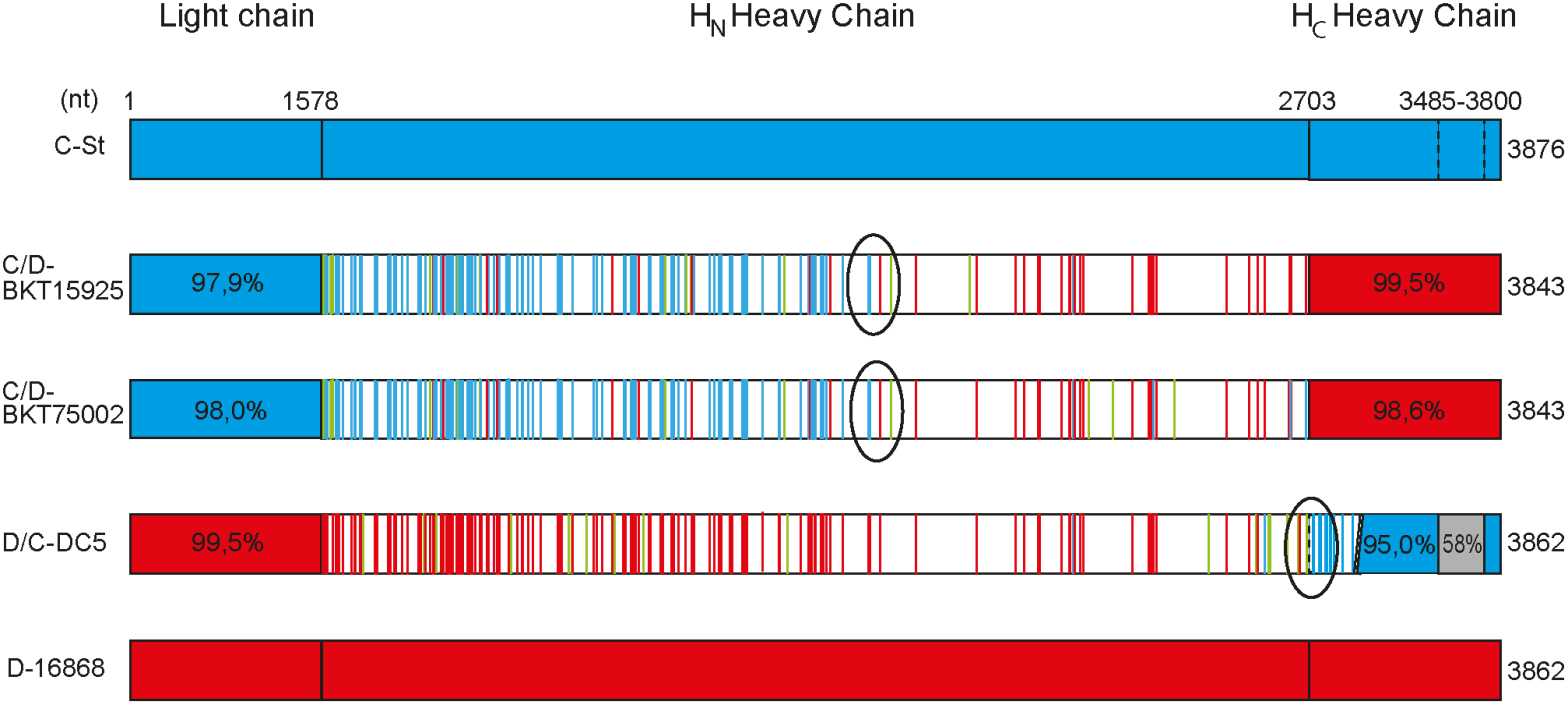
Alignment of chimeric *bont* sequences in comparison with non-chimeric sequences. Chimeric sequences are aligned against non-chimeric sequences from type C strain C-Stockholm (lineage II) and type D strain 16868 (lineage I). The chimeric sequences are from type C/D strains BKT015925 and BKT75002, and type D/C strain DC5 (all from lineage I). In the H_N_ domain, nucleotides that are conserved are not colored, whereas unique nucleotides are marked in green, nucleotides corresponding to the C-sequence are marked in blue, and nucleotides corresponding to the D-sequence are in red. The light chain and the H_C_ domain are not represented according to scale and they are colored as the closest related sequence. Estimated recombination sites are indicated.

Sequence comparisons of the *bont* genes from chimeric strains with non-chimeric strains revealed the approximate position for the probable recombination event between two *bont* genes of types C and D. For the chimeric type C/D, the recombination point was identified in the N-terminal domain of the heavy chain and for the D/C *bont* sequences in the transition between the N- and C-terminal domain of the heavy chain (Figure 4). The second recombination site could not be explicitly defined; however, it probably occurred within the *ntnh* genes which, when compared with sequences from all group III subtypes, show more than 97% nucleotide sequence identity. The sequences corresponding to type D in the *bont* genes of both chimeric serotypes were very similar (>98%) to the type D strain 16868 in lineage Ia (Figure 4). No type C reference sequence was available from lineage I. The type C reference sequence from lineage II was more diverged from the chimeric serotypes than the type D reference sequence.

### Genes coding for C2 toxin, Phospholipase C and Epsilon toxin B are carried by a subset of the plasmids in PG3, PG4 or PG6

Genes coding for the C2 toxin components (C2I and C2II) were found only on the PG3 plasmids originating from subgroup Ia of lineage I and in the subset of the PG4 plasmids derived from *C. botulinum* strains. A gene coding for a phospholipase C was also found in PG3 plasmids in lineage Ia. This phospholipase C gene was similar to a family of conserved chromosomal genes, which are annotated as gamma toxin in *C. novyi* type A and beta toxin in *C. novyi* type B and *C. haemolyticum*. The relationship between these different phospholipase C genes is shown in Table 2. The amino acids predicted to be involved in zinc-binding were conserved in all the sequences [11]. *C. botulinum* plasmids in PG4 carried a single copy of an epsilon toxin B gene, and PG6 plasmids carried two tandem located epsilon toxin B genes (38–40% amino acid identity between corresponding genes of the two plasmid groups). In PG6, these tandem genes were located just downstream a site-specific recombinase, which was also found, albeit on a different location, in PG4 (∼90% amino acid identity). Apart from the recombinase and the epsilon toxin, plasmid groups PG4 and PG6 did not share any other genes.

**Table 2:** Comparison of phospholipase C encoding genes in *C. novyi sensu lato* genomes.

Lineage	Species	Strain	Amino acid identity (%) to*C. haemolyticum* beta toxin(AF525415)	Location
Ia	*Clostridium botulinum*	BKT015925	90	chromosome
	*Clostridium botulinum*	BKT015925	74	p3BKT015925
Ib	*Clostridium botulinum*	BKT75002	92	chromosome
II	*Clostridium botulinum*	1873	94	chromosome
	*Clostridium novyi* type B	NCTC 9691	99	chromosome
	*Clostridium haemolyticum*	NCTC 8350	100	chromosome
III	*Clostridium botulinum*	C-Eklund	57	chromosome
	*Clostridium novyi* type A	4552	57	chromosome
IV	*Clostridium botulinum*	It1	57	chromosome
	*Clostridium novyi* type A	BKT29909	57	chromosome

Comparison of phospholipase C genes found in *C. novyi sensu lato* genomes to the characterized beta toxin gene (accession number AF525415) of *C. haemolyticum* strain 7170. Chromosomal phospholipase C genes were found in all strains in this study and this table shows only representatives of the different species in the different lineages.

### The *C. novyi* alpha toxin resides within a group of plasmids connected by phage movements

**Figure 5:**
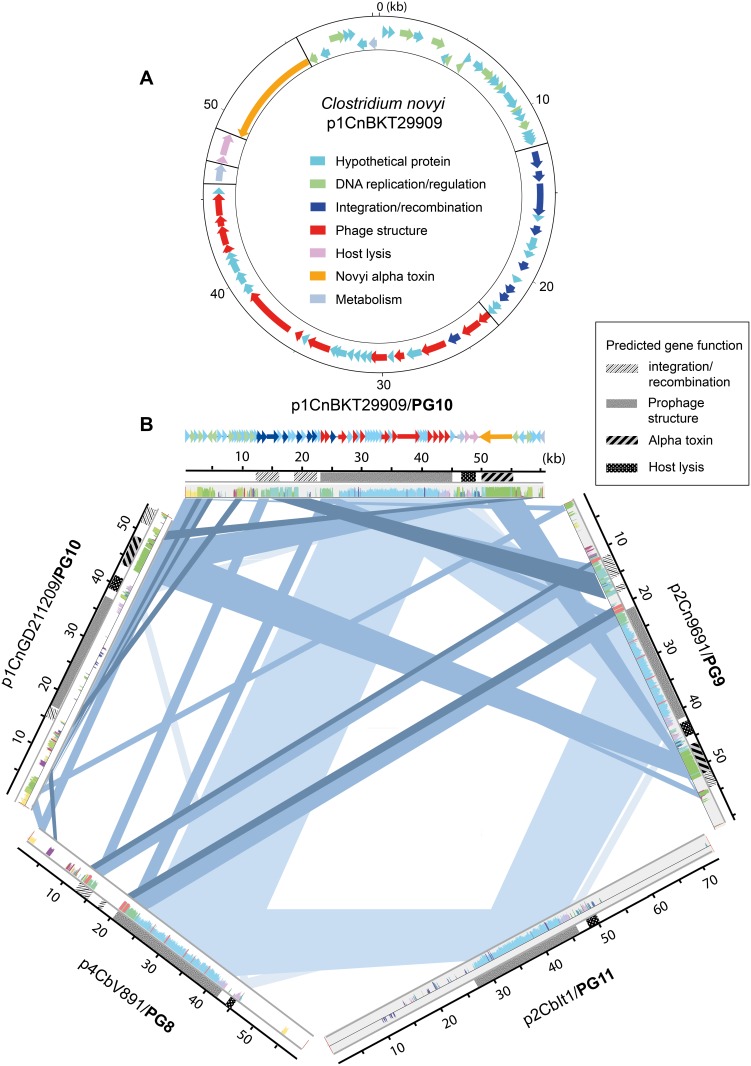
The modular novyi alpha toxin encoding-prophage and a comparison of PG8–PG11 prophages. **A.** Circular representation of the novyi alpha toxin-encoding prophage p1CnBKT29909 (60,360 bp) in PG10 from *C. novyi* type A strain BKT29909. Predicted genes are color-coded according to function and predicted modules are separated by black lines. **B.** Alignment between representative plasmids from PG8–PG11 (reference plasmid p1CnBKT29909). The colored segments in the alignment panels correspond to regions with similarities in at least one additional replicon. Regions of similarity (>1 kb) between two, three, four or five replicons are marked with blue bands, from dark to lighter blue. The functional classes of the predicted genes (in p1CnBKT29909) are colored as in figure 5A and functional regions involved in exchange are coded by different gray patterns.

This is the first report describing the sequence of an alpha toxin-encoding *C. novyi* phage. A circular representation of an annotated *C. novyi* type A prophage belonging to PG10 is shown in Figure 5A. Like many other double-stranded tailed phages [25], it has a modular structure, consisting of clusters of genes related to a certain biological function. The alpha toxin-encoding prophages were part of a cluster of plasmid groups, PG8–PG11, which clearly were related to each other, as seen by their shared genetic content (Figure 2). However, the shared genetic material was to a large extent likely a consequence of phage module exchange between unrelated phages (Figure 5B). Phages in these plasmid groups originated from strains of *C. botulinum* and *C. novyi* type A and type B, and existed in all four lineages. The complex relationship between five selected representative PG8–PG11 phages is illustrated in Figure 5B.

All phages in PG8–PG11 contained a module with structural phage genes (coding for tail, baseplate and head assembly proteins) of approximately 20 kb inserted downstream genes coding for proteins involved in integration and recombination. This suggests that this region is a hot spot for phage module recombination. The same conserved structural phage gene module was found in all plasmid groups PG8–PG11, but not in all phages in these groups. The phages containing ‘unique’ structural phage gene modules were p1GD211209 and p3BKT12695 of PG10, and p2BKT12695 of PG11. Interestingly, no matter which structural phage gene module they contained, all phages of PG8–PG11 carried a related lysis- and lysogeny-regulating module. It consisted of one or two genes controlling bacterial lysis and phage release (a holin and a phage lysine) and was located downstream the phage structural gene module.

The gene coding for the alpha toxin was found in *C. novyi* strains in two of the plasmid groups, PG9 and PG10, and at approximately the same position, just downstream the lysis- and lysogeny regulating modules. The novyi alpha toxin amino acid sequence was highly conserved, even between sequences from strains of the different types, A and B (99% identity). It is likely that the two mentioned conserved modules carried the alpha-toxin gene with them when they moved between phages of the lineage I–II and lineage III–IV branches. In PG9, two mobile elements were found downstream the alpha toxin gene: one transposase (ISCbo10) and one site-specific recombinase. This is further evidence that the exchange and incorporation of modules in this phage group has been aided by IS elements and recombination enzymes. In addition to modular exchange, these phages have likely evolved by smaller deletions and insertions, given the size and genetic variations that exist in their homologous modules.

## Discussion

### The relationship between phylogenomic lineages and species

As with most pathogens, the three species *C. botulinum (group III), C. novyi* and *C. haemolyticum* are defined by their ability to cause a certain disease. These diseases are directly dependent on a few key virulence factors (the botulinum neurotoxin for *C. botulinum* and combinations of the alpha, beta and gamma toxins for *C. novyi* and *C. haemolyticum*). Most of the virulence genes are carried on plasmids or phages, which upon horizontal movements can transfer disease-causing properties. Our genomic comparison of strains representing these three different species revealed four separate lineages. In this collection of strains, which was selected to represent maximal genomic, plasmidomic and geographic diversity, the species designations correlated only modestly with the lineage formations. This observation can be explained by the mobility of toxin-encoding plasmids between lineages. Still, most plasmid sequences mimic the phylogeny of the whole genome sequences, indicating that horizontal movements of plasmids between lineages are infrequent or transient. Our analysis shows that it is primarily the bacteriophages that cross the lineage boundaries.

Previous phylogenetic studies have shown that a strain of one of the three species may be genetically more closely related to strains from one of the other species than to strains from its own species [4], [5]. This is reflected in lineage II, which consists of all three species. Thus, lineage II is the least specialized lineage, capable of hosting several different toxin-encoding plasmids and phages. This lineage also contains the majority of the isolates older than 50 years, which suggests that it was once more commonly found than it is today. PFGE subtyping of the strains from European samples that have been isolated at our institute during the last five years show that lineage I, and specifically subgroup Ia, dominates today (38 strains corresponding to pulsotypes of lineage Ia and 4 of lineage Ib). The success of this lineage is likely connected with the high frequency of chimeric botulinum toxin types found in it. Our data suggest that this lineage has developed as *C. botulinum*-specific. It has spread rapidly during the last decade as a consequence of the introduction of the chimeric toxin forms, which seem to be more toxic to their hosts than the non-chimeric ones [26]. The few strains that make up lineage III indicate that it is a less common lineage; the strains can host both botulinum toxin- as well as novyi alpha toxin-encoding phages. In addition, strains from this lineage are prone to losing their toxin-encoding phages, which may explain why this lineage is rarely found in connection with disease outbreaks. The only toxin-encoding phage for which we have a sequence from this lineage was most similar to corresponding phages from lineage I – thus it has likely been horizontally acquired from the latter. It is possible that lineage III only transiently carries either of the toxin-encoding phages. Lineage IV has evolved towards strains that we classify as *C. novyi* type A. We also found one *C. botulinum* strain in this lineage, but it had lost its toxin-encoding phage, which suggests that BoNT-encoding phage transductions in this lineage are transient occurrences.

Overall, the strains in each lineage have widely diverse origins and cannot be assigned to a restricted geographical location or to a certain animal host. All four lineages contain strains that were isolated during a period of 50–90 years, which indicates that the different phylogenomic groups have existed for a long time. We believe that these four lineages represent the main population of *C. botulinum* group III, *C. novyi* and *C. haemolyticum,* but it is possible that there are additional lineages in *C. novyi sensu lato* that are rarely associated with diseases of importance to mammals.

### Plasmids and circular prophages

Strains identified as *C. botulinum* group III, *C. novyi* type B or *C. haemolyticum* were discovered to contain a large number of plasmids or circular prophages (between three and five each), whereas strains identified as *C. novyi* type A contained only one, i.e., the alpha toxin-encoding phage. The genetically less related, but more extensively studied, physiological groups of *C. botulinum* (group I and II) have also been reported to carry plasmids [27], [28], [29], [30], but not more than two in each strain and only seven different plasmids have been identified so far in them.

The plasmids identified in *C. novyi sensu lato* were categorized into 13 different groups, on the basis of their replication and partitioning machineries, and the amount of shared genetic material. In most groups, at least one conserved replication, partitioning or plasmid maintenance gene can be found that also is unique for that group, which in addition to plasmid sequence similarity supports the division of the plasmids into these groups. However, there are probably further components of the replication and partitioning machinery that we were not able to annotate using the available reference databases. Some plasmids represent regular plasmids whereas others are circular prophages. The circular prophages are either autonomous phages that have incorporated themselves into plasmids, or prophages that have picked up plasmid replication genes. Some of the plasmid groups have putative replication and partitioning genes with similarities to those found in other plasmid groups. As a consequence, some plasmid groups could be fully or partially incompatible. For example, plasmid groups PG1 and PG2 have related partitioning genes, and this could be a contributing factor to the high instability of the BoNT-encoding phages in PG1. However, in general the prophages were more unstable than the regular plasmids. Conjugation of large plasmids in Clostridium species has been reported, e.g., in *C. botulinum* group I and II [31], but our data suggest that phage transductions are the most important factor facilitating lateral gene movement in *C. novyi sensu lato*. Generally, plasmid movements over the lineage boundaries were concluded to be infrequent events as the plasmid sequence phylogeny correlated with the lineage division. However, the relatively low amount of shared genetic material among some plasmids of the same plasmid groups and the sharing of genes among otherwise unrelated plasmid groups is evidence that there is an on-going re-arrangement of plasmids in *C. novyi sensu lato*.

Plasmid groups PG1 and PG6–12 consist of extrachromosomal circular prophages. The BoNT-encoding phages of PG1 and the novyi alpha toxin-encoding phages of PG9 and PG10 have been previously described as non-integrative lysogenic bacteriophages and experiments have shown that they can infect other *C. botulinum* or *C. haemolyticum* strains [32], [33], [34]. Lysogeny and cross-infection by *C. novyi sensu lato* bacteriophages probably occur also in their natural life cycles, e.g., in the soil or in the intestinal tract of animals. In this study we present several examples of such natural phage cross-infections between lineages. First, there are the *C. botulinum* strains in lineage III and IV, which are likely the results of infections by PG1 phages from lineage I. Second, there are the phages of PG8–PG11, including the novyi alpha toxin-encoding phages, which demonstrate a more complex result of bacteriophage movements. In this case, phages move between strains and exchange modules of functionally-related genes, probably through integrase-mediated site-specific recombination. This explains why these plasmid groups are more phylogenetically disorganized than the rest of the plasmid groups. Last there is PG12, which is also subjected to modular recombinations. Here however there are divergent modules containing genes coding for phage structural proteins that have been incorporated into phages containing the same type of DNA segregation module.

### Gene transpositions and recombination events

Both the *C. novyi* type A and *C. botulinum* group III genomes have been reported to contain an abundance of insertion sequences [6], [18]. Of the genomes analyzed in this study, the highest abundance of IS elements was found in lineage I, and especially in Ia. Subgroup Ia also contained the highest number of predicted virulence genes; these show a higher genetic and localization variation than virulence genes in the other lineages. In the *C. novyi sensu lato* plasmidome, we occasionally found IS elements adjacent to toxin genes. This could be a coincidence, but it may also be a remnant of a toxin-gene transposition event. The novyi alpha toxin gene is found in two different plasmid groups, probably as a consequence of a combination of phage and IS element movements. The lateral movements of the novyi alpha toxin gene have led to the formation of the two different *C. novyi* types, A and B. Furthermore, a putative homologue to the epsilon B toxin genes found on *C. botulinum* PG4 plasmids is localized on PG6 plasmids next to a site-specific recombinase, indicating that another toxin gene transposition event has occurred.

Transposons have previously been suggested to have been involved in the transfer of the *bont* gene cluster between strains in *C. botulinum* groups I and II [29], [35]. A truncated form of an IS6-family element (ISCbo1) has been previously located between *orfX* and *botR* in the *bont* cluster of *C. botulinum* group I strains [35]. Interestingly, we found the complete open reading frame of the same element downstream the *bont* cluster in type D strain 16868, which may support a role for this specific IS element in the lateral transfer of the *bont* cluster. A PG1 phage lacking the *bont* cluster was found in a *C. haemolyticum* strain, suggesting that this phage represents a genetic variant that once was the ancestor from which the botulinum toxin-encoding phage arose. The *C. botulinum* group III strains examined here contained a highly conserved *bont* gene cluster. In *C. botulinum* groups I and II, each serotype is divided into several subtypes, defined by *bont* genes differing by at least 2.6% amino acid identity [36]. By the same standards, the group III serotypes C, C/D, D and D/C only account for one subtype each. The higher conservation of the *bont* cluster in *C. botulinum* group III genomes in comparison with *C. botulinum* group I and II genomes, suggests that the *bont* cluster incorporation into the botulinum phage ancestor is the consequence of a single and more recent insertion event.

Lineage I contains the majority of the chimeric *bont* sequences, and it is likely that the recombination events took place in this lineage. The chimeric *bont* genes are probably the result of co-infections of two *bont*-encoding phages of different serotypes, where homologous recombination events have led to the chimeric *bont* genes. Using alignments, we could approximately identify the recombination sites in the chimeric *bont* sequences in two different positions, one for each serotype (C/D and D/C). The second recombination site is probably located in the conserved *ntnh* gene upstream the *bont* cluster, which has previously been implicated in recombinations [37], [38]. This means that the type C/D *bont* gene is a recombination product between an existing type D BoNT-encoding phage and a *bont* gene from a transiently co-infected type C phage, and the other way around for serotype D/C.

### *C. novyi sensu lato*: a complex group of entwined species

The different species in *C. novyi sensu lato* are identified by their pathogenic traits and the diseases they cause. Traditionally, all strains with the ability to produce botulinum neurotoxin are called *C. botulinum*, hence the species can be identified from the presence of the botulinum neurotoxin gene. *C. novyi* causes gas gangrene and black disease and is identifiable by the production of the novyi alpha toxin. Additionally the chromosomally encoded phospholipase C (beta toxin) can be used to differentiate *C. novyi* type B from type A, which produces a non-toxic phospholipase C (gamma toxin). The laboratory diagnosis of black disease (caused by *C. novyi* type B) and bacillary hemoglobinuria (caused by *C. haemolyticum)* is usually made on the basis of an analysis of the 16S–23S spacer region or fluorescent antibody detection. Unfortunately, neither technique is able to differentiate the two species [11], [39]. Furthermore, the pathogenecity of *C. haemolyticum* is attributed to the beta toxin, which is very similar to the phospholipases produced by *C. novyi* type B and *C. botulinum* group III strains. Thus, a *C. novyi* type B strain or a *C. botulinum* type III strain that loses the alpha toxin/BoNT-encoding phage is taxonomically transformed into a *C. haemolyticum* strain. Although it has been shown that the beta toxin gene of *C. haemolyticum* is more highly expressed than that in *C. novyi* type *B* or *C. botulinum* [9], it is speculated that sufficiently high amounts of the toxin can also be produced by *C. novyi* type B and *C. botulinum* group III strains during toxicoinfection, thereby contributing to the pathogenesis of these two species [40].

In recent years, classical bacterial systematics is being challenged by the genomics revolution, which provides new information for taxonomical consideration. In summary, we here outline the genomic complexity within the *C. novyi sensu lato* group, which contains the entwined species *C. botulinum* (group III), *C. novyi* and *C. haemolyticum.* The complexity primarily depends on phages, carrying toxin genes over lineage boundaries. Given that the phages in these species are in a close relationship with the plasmids and have to some extent evolved by modular exchange between unrelated phages, it is not surprising that they also occasionally acquire toxin genes.

## Materials and Methods

### Bacterial strains

The strains used in the study are described in Figure 1. They represent a collection comprising isolates classified as *C. botulinum* (type C, C/D, D/C or D), *C. haemolyticum* or *C. novyi* (type A or B). Strains were selected on the basis of diversity of origin (source, geographical location and year), PFGE pulsotypes, and plasmid profiles. PFGE subtyping was performed as previously described [41]. Plasmid separation by PFGE was used estimate plasmid number and sizes in each strain. Agarose plugs were made as previously described [6], except that S1 nuclease was not used as it was deemed unnecessary. Uncleaved DNA was size-distributed by PFGE in a 1.2% (w/v) agarose gel (Agarose NA, GE Healthcare, Little Chalfont, UK) and electrophoresis was performed at 14°C in HEPES buffer (16 mM HEPES-NaOH, 16 mM sodium acetate, 0.8 mM EDTA, pH 7.5). The settings were 4 V/cm for 26 hours at a switch time ramped from 0.5–15 s in a CHEF DRII apparatus (BioRad, Hercules, CA, USA). *C. novyi* type B strain 9691 and *C. haemolyticum* strain 9693 and 8350 were purchased from the National Culture Type Collection (NCTC) of the Health Protection Agency, Great Britain; *C. novyi* type B strain ATCC 27606 from the Leibniz Institute-DSMZ (DSM number 5566); and *C. botulinum* type D strain 7970 from the Culture Collection, University of Göteborg, Sweden. *C. novyi* type A strains NCTC 538, 4552, 4540 and 4570 were kindly provided by Ian Poxton (University of Edinburgh, Great Britain) and *C. novyi* type A strain GD211209 and *C. botulinum* type D strain 16868 by Miriam Koene (CVI, the Netherlands). Remaining strains were isolated by the authors as previously described [42]. Strain Sp77 was isolated in collaboration with Ibone Anza Gomez (IREC, Spain), strain It1 in collaboration with Cedric Woudstra (ANSES, France), and K25 in collaboration with Beying Yong (QIA, Korea). No experimental animals have been used in this study. Samples were only collected from dead animals in natural disease outbreaks and no animal was killed for the purposes of this study.

### Preparation of genomic DNA

Bacterial strains were cultured overnight in anaerobic jars (Merck, Darmstadt, Germany) at 37°C in 9 ml pre-reduced TPGY broth [41]. Cells were harvested by centrifugation at 3000×*g* for 15 min before DNA extraction. Genomic DNA was purified using the Master Pure Gram Positive DNA Purification Kit (Epicentre, Madison, USA). The DNA concentration was measured with a Qubit Fluorometer (Life Technologies, Carlsbad, USA) and the quality of the DNA was analyzed on a NanoDrop 2000 (NanoDrop Technologies, Wilmington, USA) and by gel electrophoresis. A Bioanalyzer 2100 (Agilent Technologies, Santa Clara, USA) was used to determine DNA concentration and size distribution during library preparation.

### Genome sequencing and *de novo* assembly

High-throughput sequencing of genomic DNA of 9 strains, Sp77, 4540, 4570, 4552, NCTC 538, BKT29909, GD211209, It1 and BKT12695 was performed using the Roche 454 Genome Sequencer with FLX+ chemistry (Roche Applied Science, Mannheim, Germany) at the Science for Life Laboratory (SciLife Lab, Stockholm, Sweden). DNA from the same strains was also prepared into TruSeq libraries and paired-end sequenced (2×100 bp) on Illumina HiSeq 2000, using 300 bp inserts, also at the Science for Life Laboratory. Libraries of strains BKT75002, BKT2873, 16868, CCUG 7971, NCTC 9693, NCTC 8350, NCTC 9691, ATCC 27606, K25 and DC5 were prepared using the Nextera XT DNA Sample Preparation kit (Illumina, San Diego, USA) and paired-end sequenced (2×250 bp) on an Illumina MiSeq Benchtop Sequencer, with approximately 300 bp inserts. Library preparation and sequencing were performed as recommended by the manufacturer. Approximately 5 µg genomic DNA was used from each strain for library preparations for 454 FLX+ sequencing and Illumina HiSeq sequencing, whereas 1 ng was used for Nextera XT libraries subjected to Illumina MiSeq sequencing. The 454 FLX+ sequences were *de novo* assembled with the GS assembler (Newbler, Roche Applied Science, Mannheim, Germany). Approximately 1–3 million Solexa reads (Table S1), derived by the Illumina MiSeq technology, were extracted and *de novo* assembled using SPAdes (version 3.0.0) [43] and the MIRA software package (version 3.4.0) (http://sourceforge.net/projects/mira-assembler). Paired-end reads for *de novo* assembly in MIRA were first pair-wise assembled using COPE [44]. Contigs larger than 500 bp were kept for analysis. A detailed specification of the assemblies is presented in Table S1.

### Identification of plasmid contigs

Several methods were applied to identify and extract contigs belonging to plasmids in order to identify the total plasmid content in each strain and to be able to close plasmid sequences, including electrophoretic plasmid profiling, genome alignments and curation experiments.

Plasmid separation by PFGE was used to estimate plasmid number and sizes in each strain and was performed as described above. Plasmid sizes were analysed with BioNumerics 6.5 (Applied Maths, Ghent, Belgium) using Lambda Ladder PFG Marker (New England BioLabs, Ipswich, USA) for the reference system size standard.Genome alignments were made with the MUMmer software (version 3.22) [45] to identify: contigs matching to reference plasmids, or contigs not matching to previously sequenced chromosomes or reference plasmids (i.e., they belonged to an unknown plasmid).

Curation experiments were performed on strains BKT015925, NCTC 9691, NCTC 9693, NCTC 8350 and ATCC 27606. The strains were cultured overnight as described above and then diluted (1∶100) after which they were cultured until OD600 until they reached exponential growth phase. At an OD600 of 0.5 the cultures were diluted again (1∶100), and heat treated (1.5 min at 70°C). After heat treatment, the samples were incubated overnight, and the same method was applied again, but this time the last dilution was made in TPGY broth supplemented with novobiocin (50 ug/ml, Sigma Aldrich, St. Louis, USA). Novobiocin slows down plasmid replication and is used to cure plasmids [46]. Samples were incubated anaerobically overnight at 37°C and plated the following day on McClung Toabe agar. Two colonies of each strain were selected and genomic DNA was prepared as described above. The DNA samples were sequenced with the Illumina MiSeq technology and assembled, both as described above. Assemblies were compared with the primary assemblies of corresponding strains by MUMmer genome alignments and contigs not existing in the new assemblies were identified as plasmid DNA.

### Closing plasmid sequences

Gaps between plasmid contigs were closed with help of the Consed package [47]. Illumina HiSeq reads (50x coverage) were mapped onto 454-assemblies in Consed and used for error correction of closed plasmid sequences.

### Nucleotide sequence accession numbers

Sequenced genomes where submitted to Genbank as whole-genome shotgun assemblies and accession numbers are given in Table S1. The genome sequences of *C. botulinum* strains BKT015925 (accession number CP002410-15), 1873 (accession number ACSJ00000000), and C-Eklund (accession number ABDQ00000000) were retrieved from the NCBI database (http://www.ncbi.nlm.nih.gov/genbank/). Reference plasmids were also collected from the NCBI database. They were: p1BKT015925-p5BKT015925 from *C. botulinum* type C/D strain BKT015925 (accession numbers CP002411-15); C-St phage and p6CbCt from *C. botulinum* strain C-Stockholm (accession numbers AP008983 and AESA00000000); and pCLG1 and pCLG2 from *C. botulinum* type D strain 1873 (accession numbers CP001659 and CP001660).

### Gene annotation

Genes in completed plasmids were defined with Glimmer (version 3.02) [48]. The annotation process was handled with Artemis software [49]. Well-conserved genes were automatically annotated from reference plasmids but less certain annotations were manually assigned. The length, identity, and coverage of the sequences of the subject-versus-query were inspected. The whole genome shotgun assemblies were annotated by the NCBI Prokaryotic Genome Annotation Pipeline (https://www.ncbi.nlm.nih.gov/genome/annotation_prok/).

### Sequence comparisons

The Gegenees software (version 1.1.5) was used to perform the phylogenomic analyses [50]. Briefly, Gegenees uses a fragmented approach to make all-against-all BLASTN whole genome comparisons. A fragment length of 200 bp and step-size of 100 bp was used. The average scores of all fragment comparisons can be used as a measurement of overall genomic similarity and these are shown in a heat-plot. The separation into phylogenomic lineages was based on a threshold of 70% overall genomic similarity. In order to define the relative amount of shared genetic material between plasmids, the conserved sequences were defined as regions constituted by fragments scoring at least 40% of the max score. Gegenees was also used to identify conserved markers for the different plasmid groups. Plasmids were aligned for comparisons using the MUMmer software (version 3.22), the Mauve Genome Alignment Software (version 2.3.1) [51] or the Artemis Comparison Tool (version 11.0.0) [52]. BoNT-encoding genes were aligned with Clustal Omega (EMBL-EBI) using a gap penalty of 6 and a gap extension of 1 bit. Remaining sequence comparisons were performed using BLASTN or BLASTP (BLAST version 2.2.25+).

### Identification and estimation of IS element copy-number

The presence and copy-number of the IS elements characterized in strain BKT015925 have previously been defined [6]. Exactly 500,000 Illumina reads from BKT015925 were aligned against the 13 different identified IS elements and the number of reads corresponding to one copy of the element was calculated. The same number of reads was extracted from remaining sequenced genomes and aligned against the same elements. The copy-number in each genome was estimated from the number of reads aligned against each element, providing that there was coverage for the whole element.

## Supporting Information

Figure S1 **The completed plasmids.** Circular representations of the plasmids completed in this study. The circle represents, beginning with the outermost, plus strand genes, minus strand genes, variation in GC and GC skew.
(PDF)

Figure S2 **PFGE plasmid profiles.** Plasmid separation by pulsed-field gel electrophoresis was used to estimate the number of plasmids and their sizes in each strain. DNA from strain BKT015925, where a completed genome was already available, was included as reference. Lambda Ladder PFG Marker was used as a standard and a size ladder was calculated using BioNumerics 6.5.
(TIF)

Figure S3 **Similarity matrix showing the core sizes (40% normalized BLASTN score threshold) between** ***C. novyi sensu lato*** **plasmids.** The full similarity matrix corresponding to the heat plot in Figure 2, showing the relative amount of shared genetic material between plasmids of *C. novyi sensu lato.*
(TIF)

Figure S4 **Alignment of the** ***bont*** **gene cluster and surrounding genes in two strains in lineage I.** The aligned sequences are from type D strain 16868 and type C/D (subgroup Ia) strain BKT75002 (subgroup Ib). The *bont* cluster is marked in green and the complete IS element (ISCbo1) identified in strain 16868 is marked in red.
(TIF)

Table S1 **Sequencing data and assembly quality statistics.**
(XLSX)

Table S2 **Predicted presence and copy-number of IS elements in the strains.**
(XLSX)

Table S3 **Genomic properties of the plasmids identified in** ***C. novyi sensu lato*** **.**
(XLSX)

Table S4 **The result of the plasmid curation experiment.**
(DOCX)

Table S5 **Plasmid replication and maintenance genes.**
(XLSX)
