# Supplementary material for: Plasmidome Interchange between Clostridium botulinum, Clostridium novyi and Clostridium haemolyticum Converts Strains of Independent Lineages into Distinctly Different Pathogens
Source: PLoS One. 2014 Sep 25;9(9):e107777. doi: 10.1371/journal.pone.0107777 (PMC4177856; doi:10.1371/journal.pone.0107777)

Clostridium botulinum. Sp77
p2CbSp77 (PG2)
96517 bp

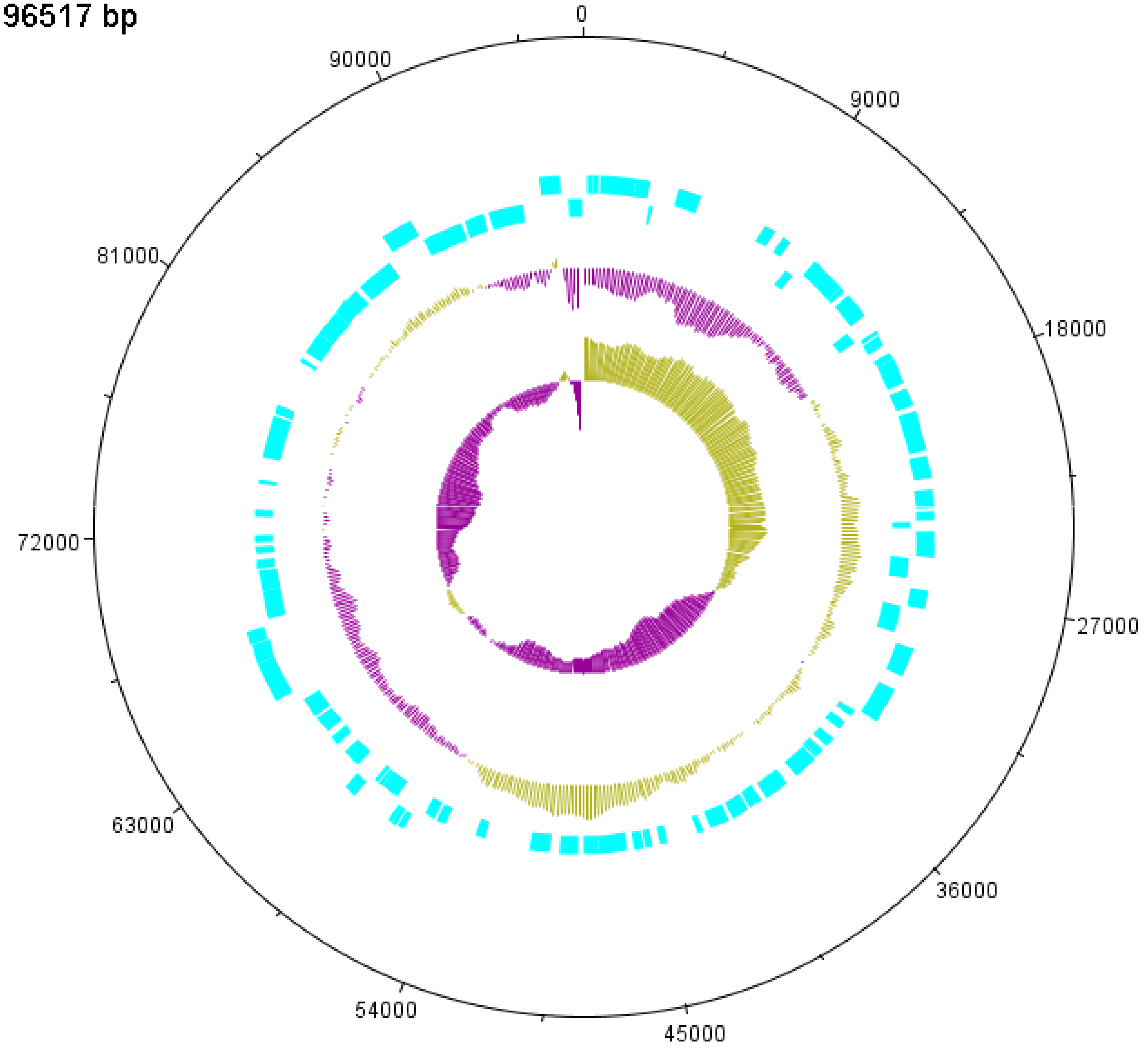

Clostridium botulinum. Sp77
p3CbSp77 (PG3)
80451 bp

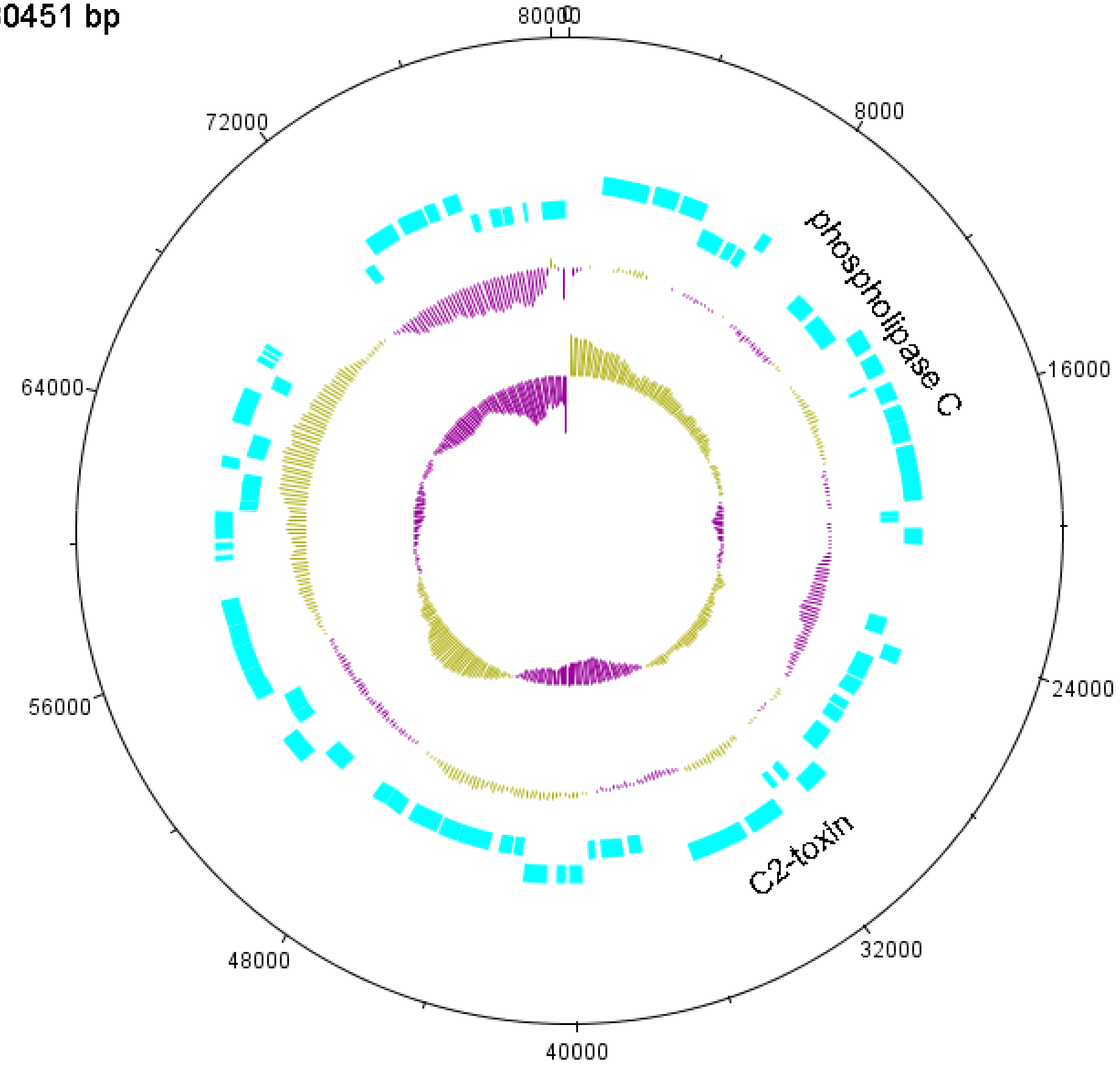

Clostridium botulinum. Sp77
p4CbSp77 (PG6)
12408 bp

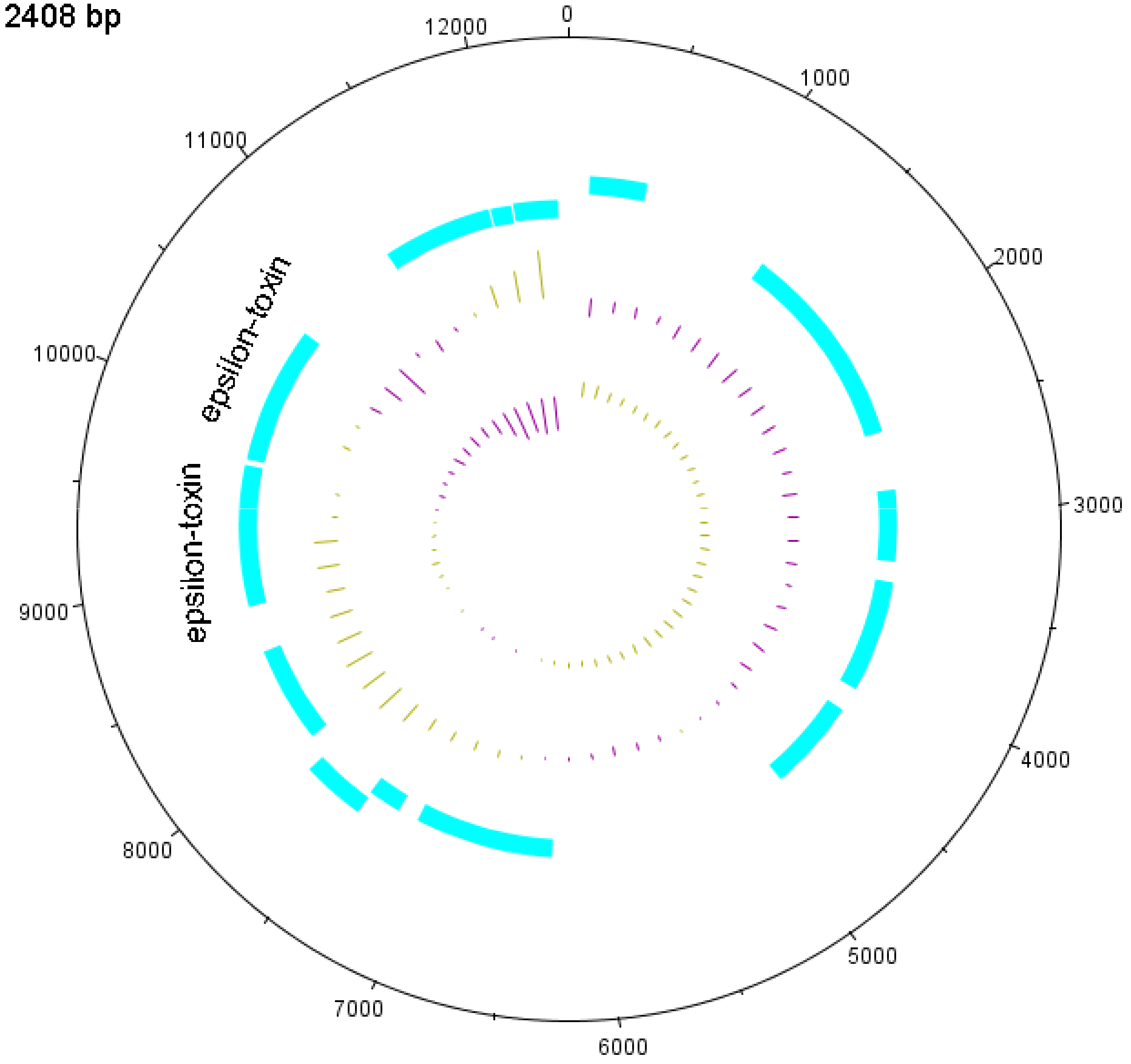

Clostridium botulinum. V891
p1CbV891 (PG1)
199891 bp

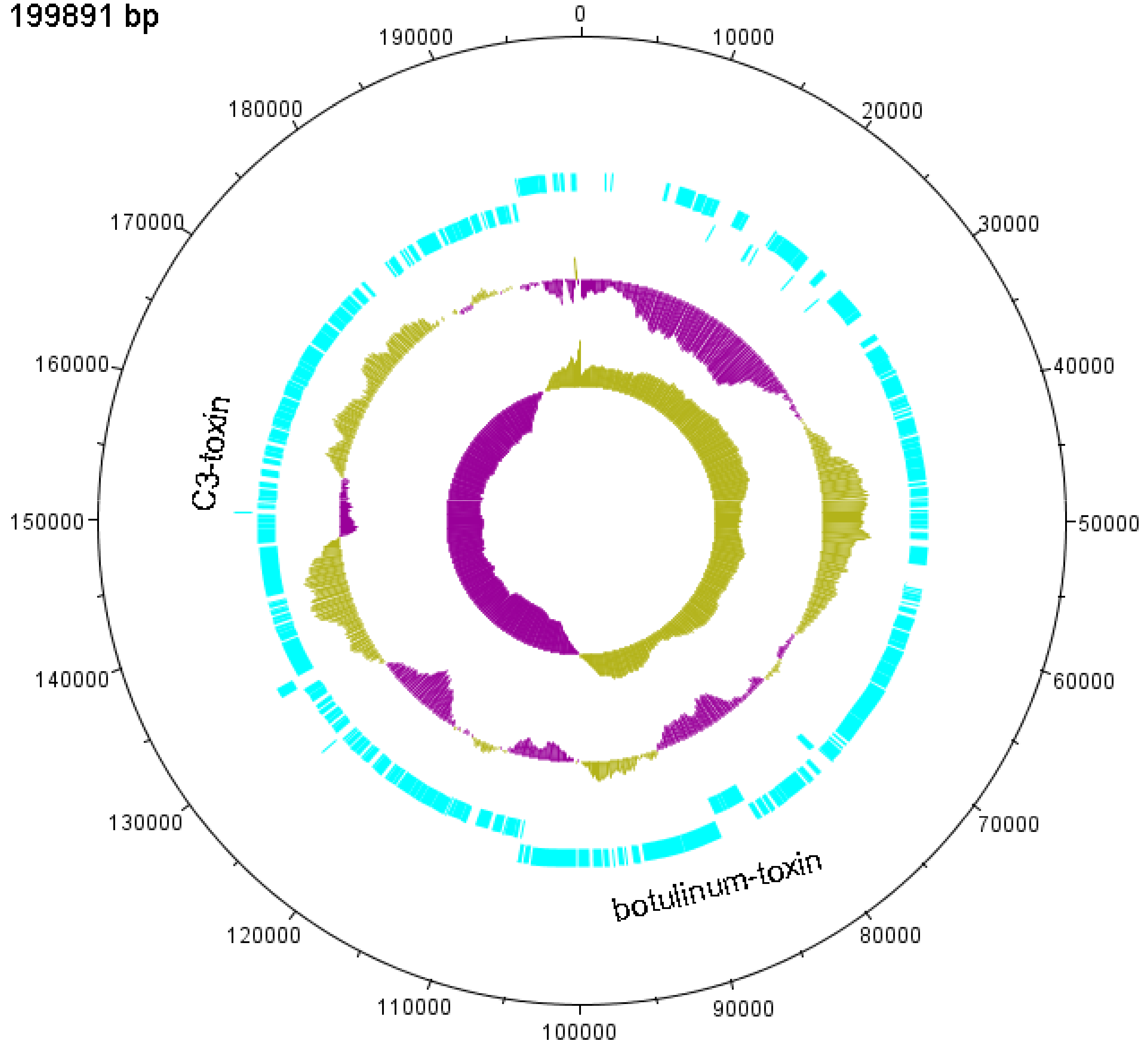

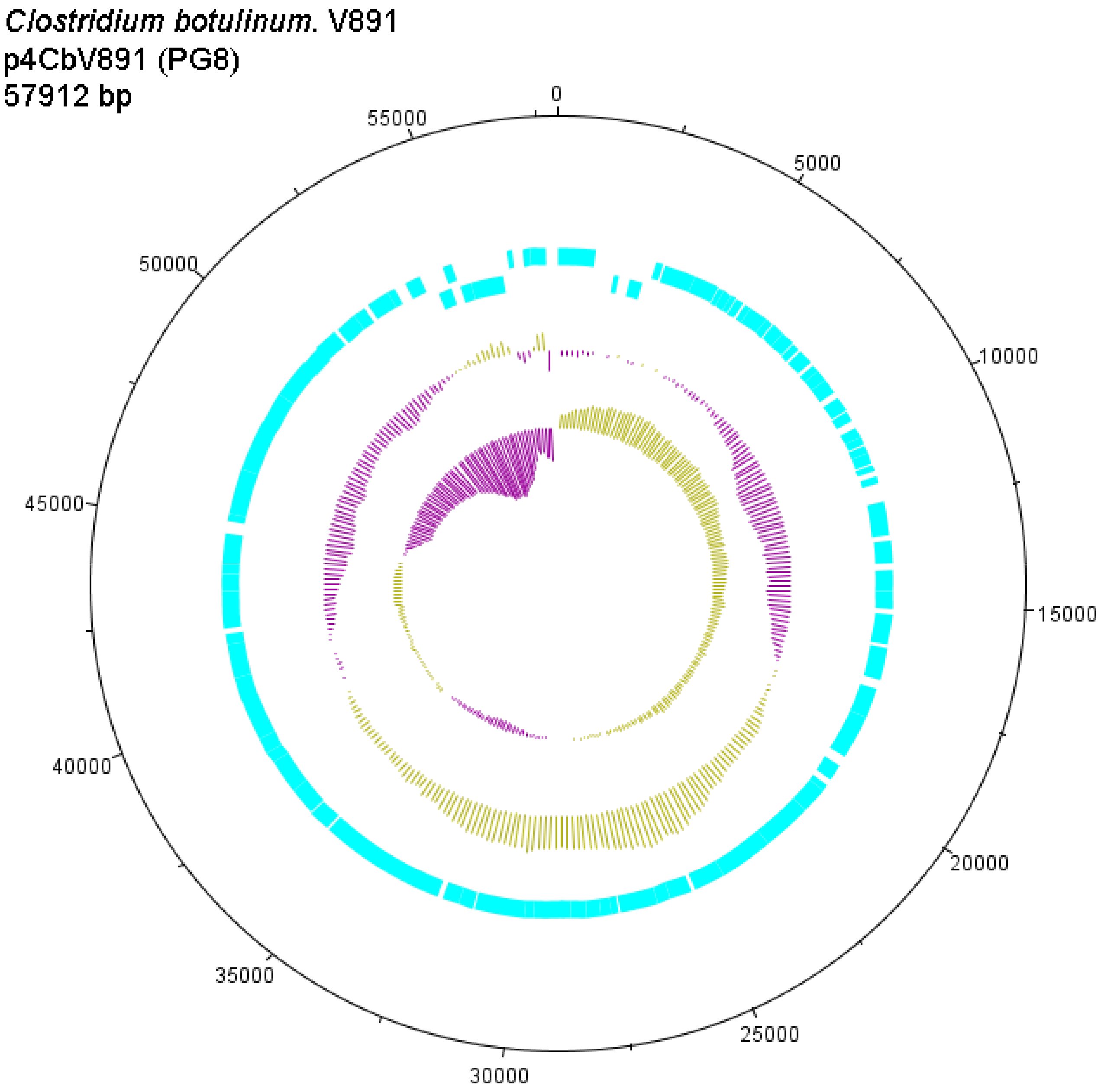
Clostridium botulinum. V891
p4CbV891 (PG8)
57912 bp
0
5000
10000
15000
20000
25000
30000
35000
40000
45000
50000
55000

Clostridium botulinum. V891
p5CbV891 (PG7)
39664 bp

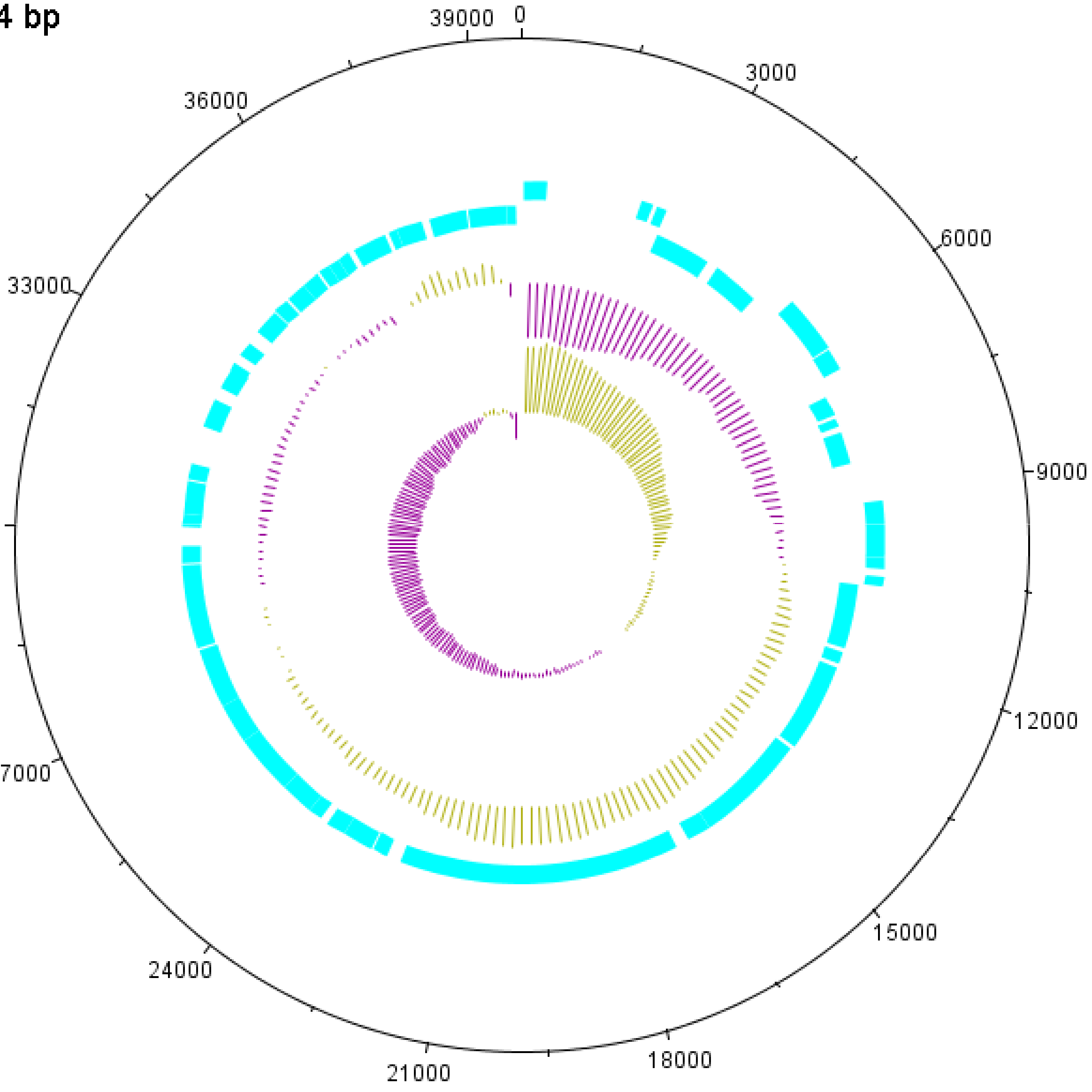

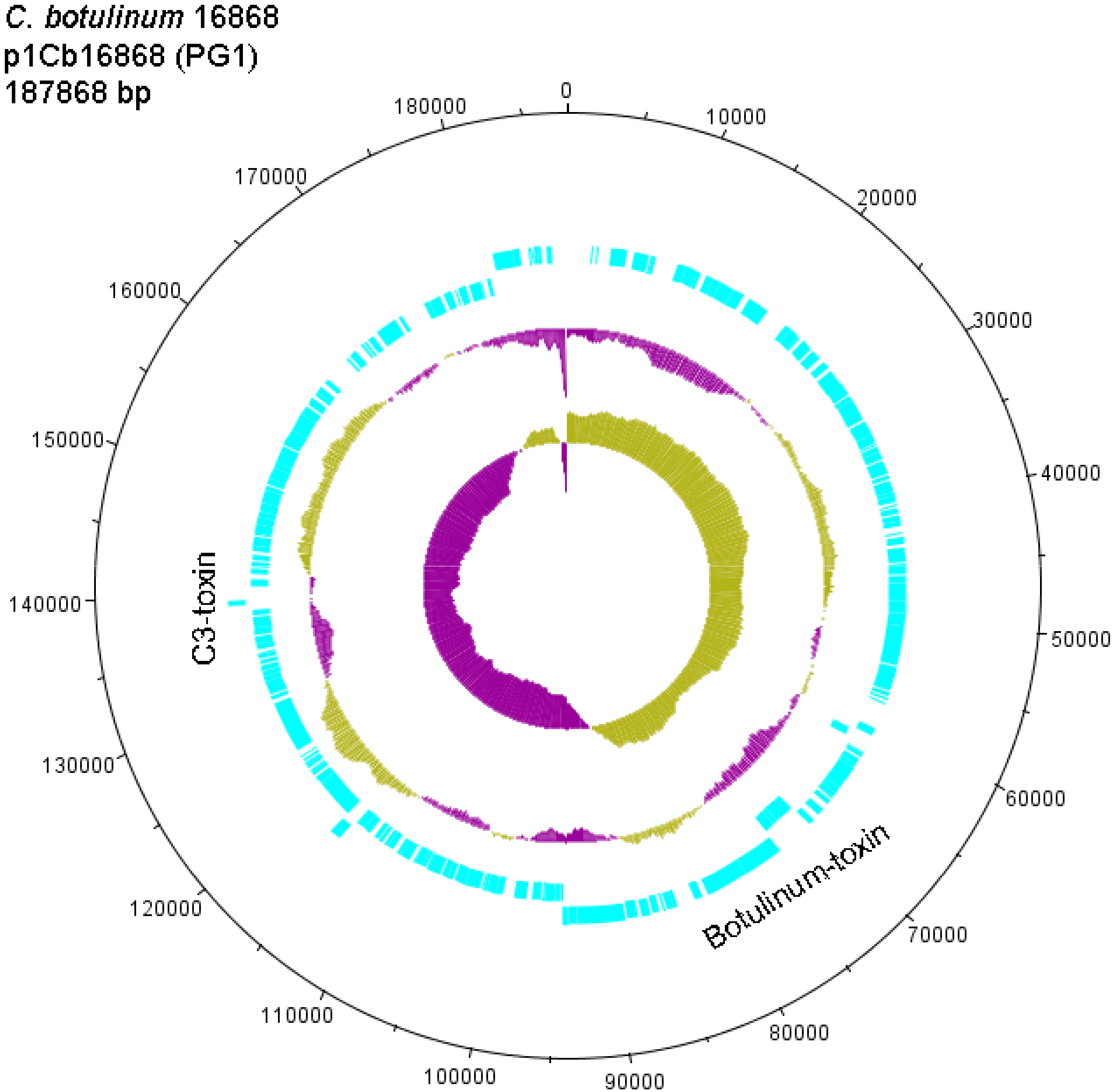

C. botulinum 16868
p1Cb16868 (PG1)
187868 bp
0
10000
20000
30000
40000
50000
60000
70000
80000
90000
100000
110000
120000
130000
140000
150000
160000
170000
180000
C3-toxin
Botulinum-toxin

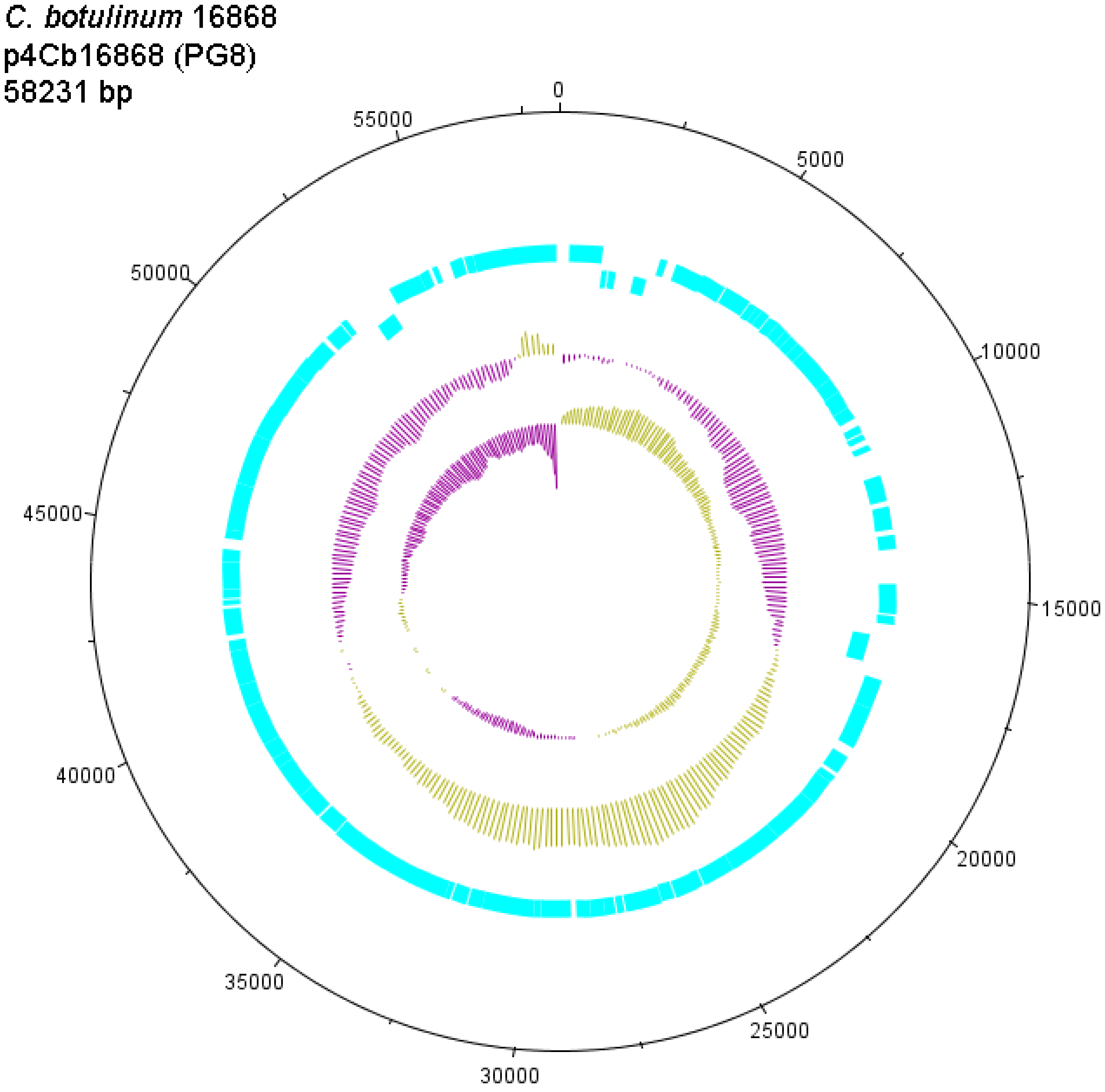

C. botulinum 16868
p4Cb16868 (PG8)
58231 bp
0
5000
10000
15000
20000
25000
30000
35000
40000
45000
50000
55000

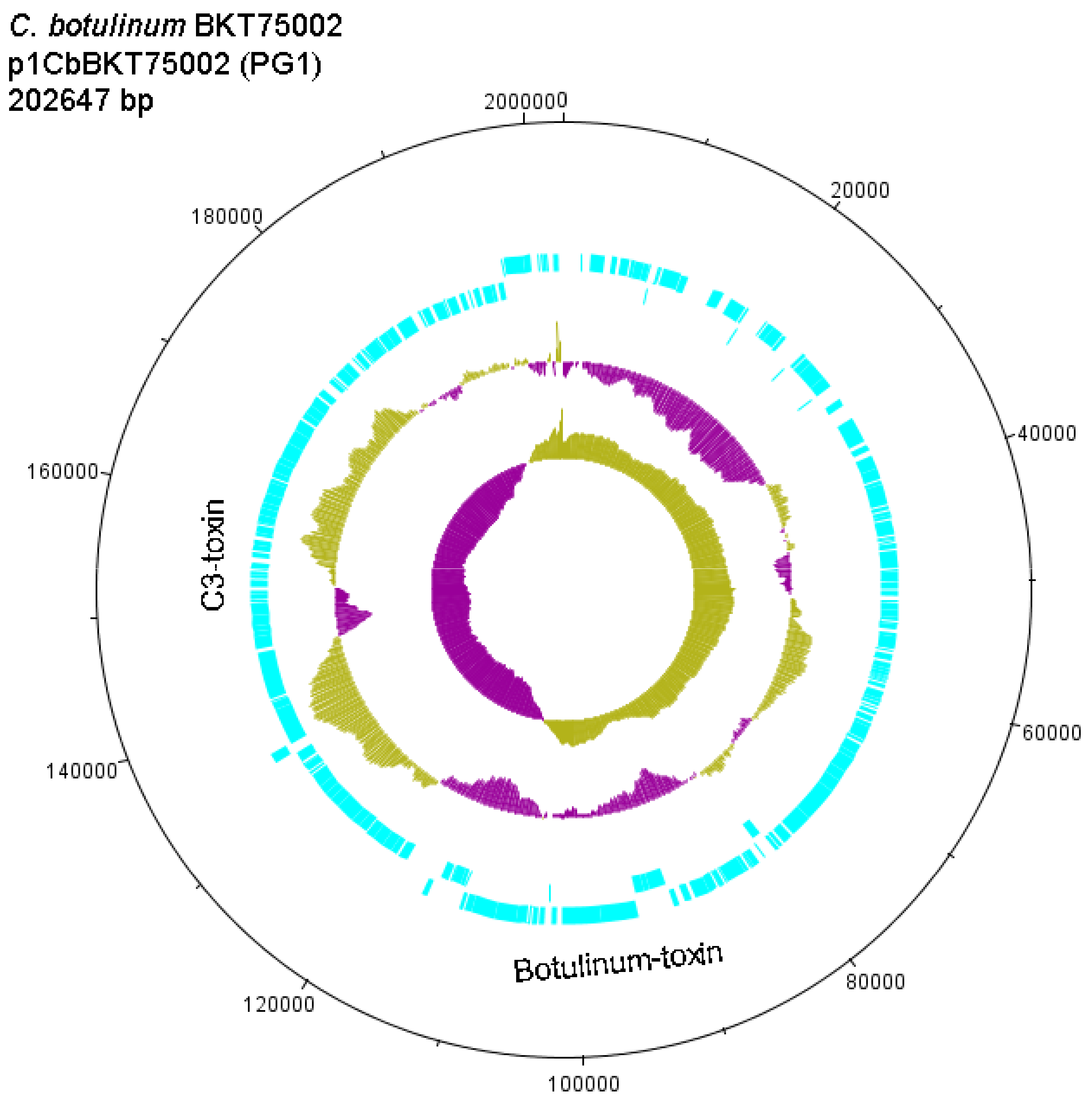
C. botulinum BKT75002
p1CbBKT75002 (PG1)
202647 bp
2000000
20000
40000
60000
80000
100000
120000
140000
160000
180000
C3-toxin
Botulinum-toxin

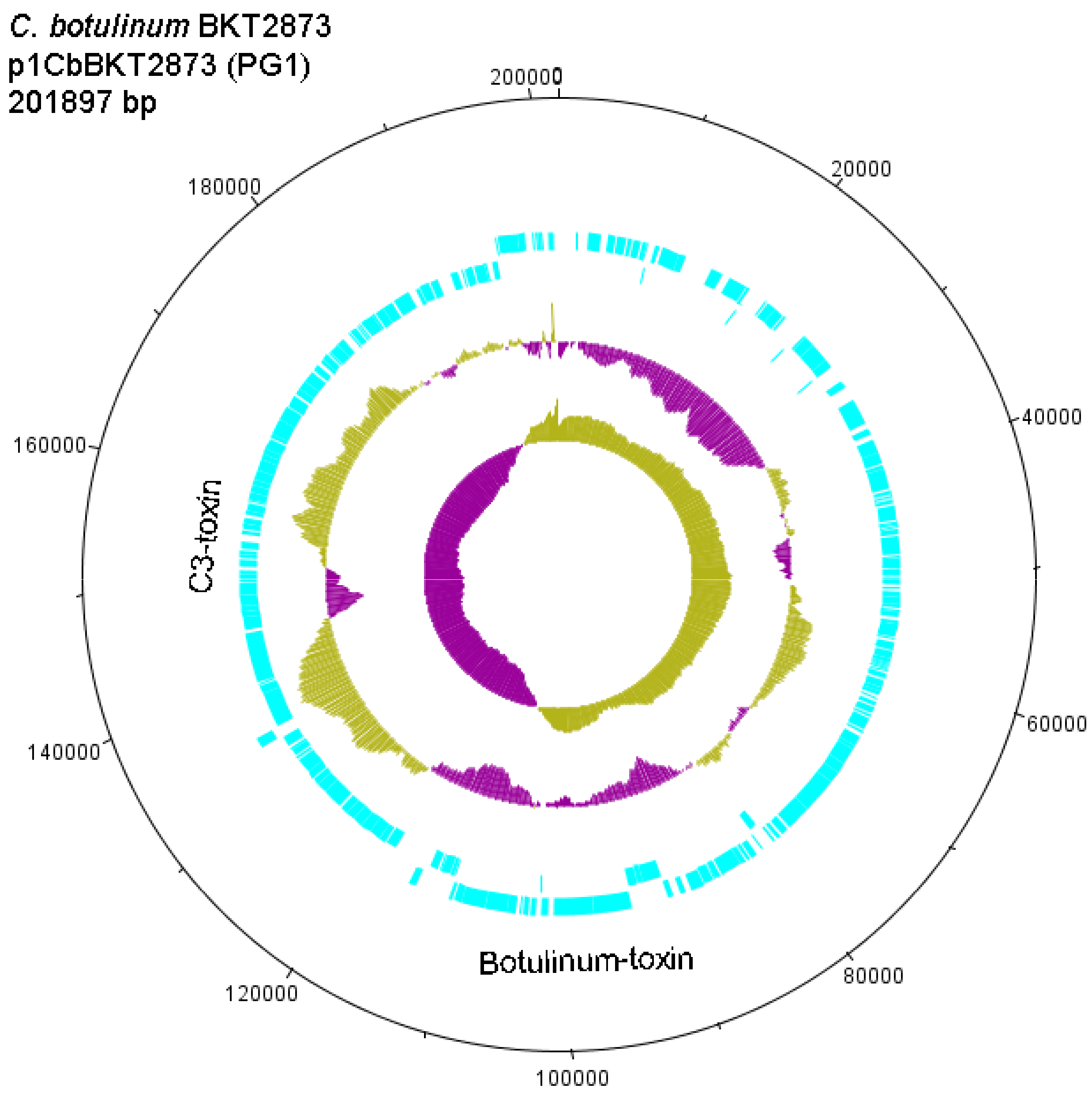

C. botulinum BKT2873
p1CbBKT2873 (PG1)
201897 bp
200000
0
20000
40000
60000
80000
100000
120000
140000
160000
180000
C3-toxin
Botulinum-toxin

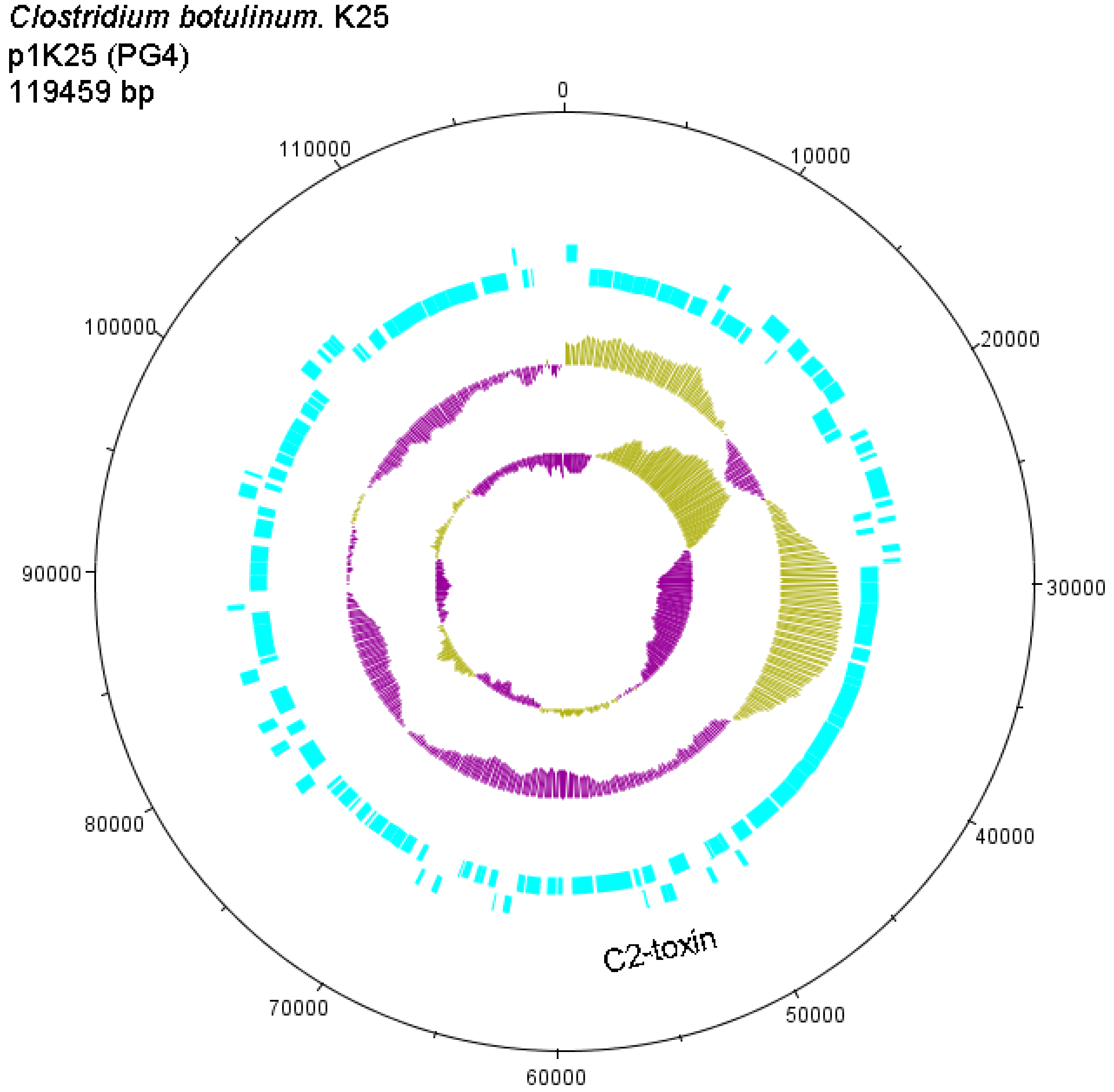
Clostridium botulinum. K25
p1K25 (PG4)
119459 bp
0
10000
20000
30000
40000
50000
60000
70000
80000
90000
100000
110000
C2-toxin

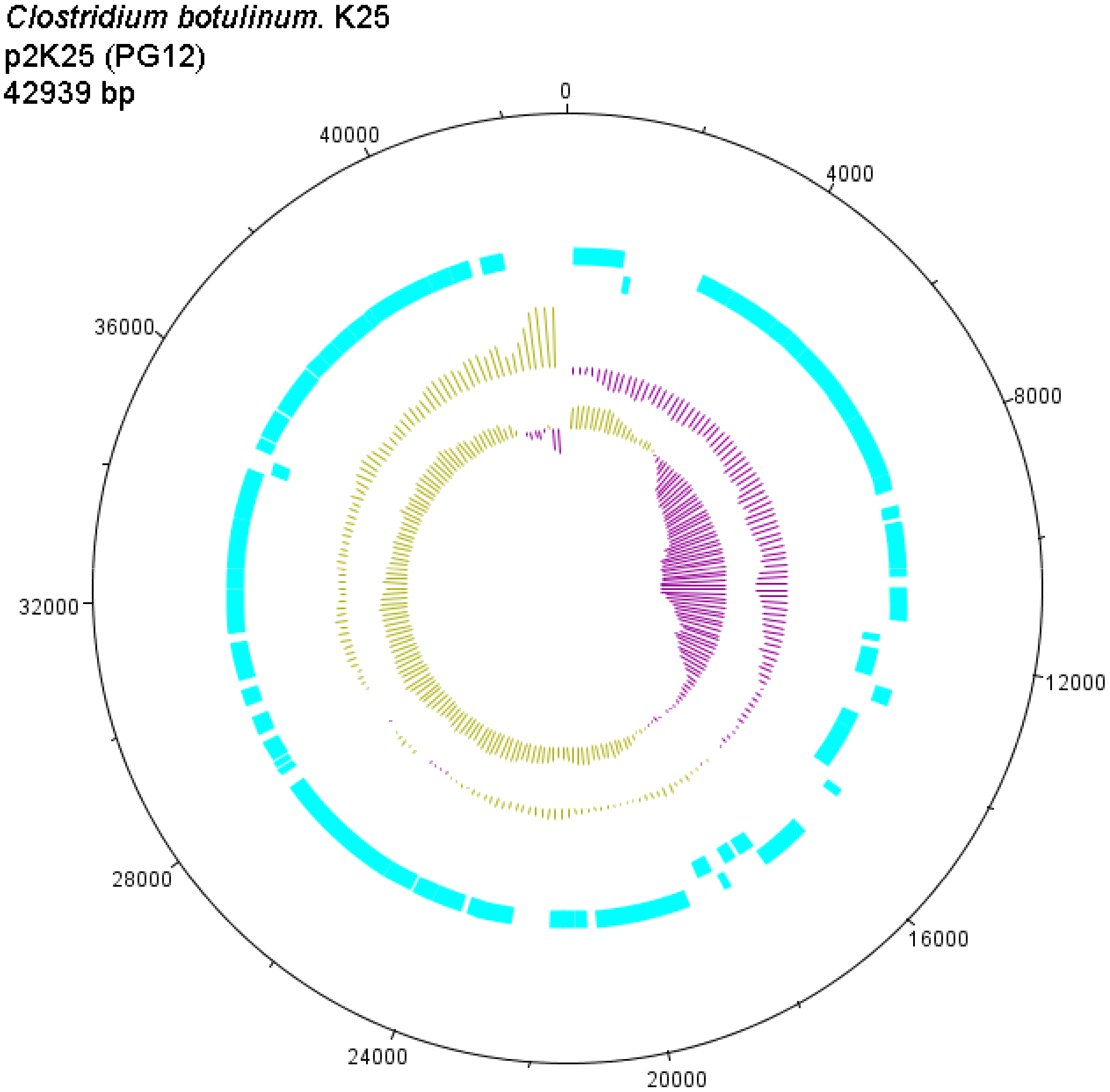

Clostridium botulinum. K25
p2K25 (PG12)
42939 bp
0
4000
8000
12000
16000
20000
24000
28000
32000
36000
40000

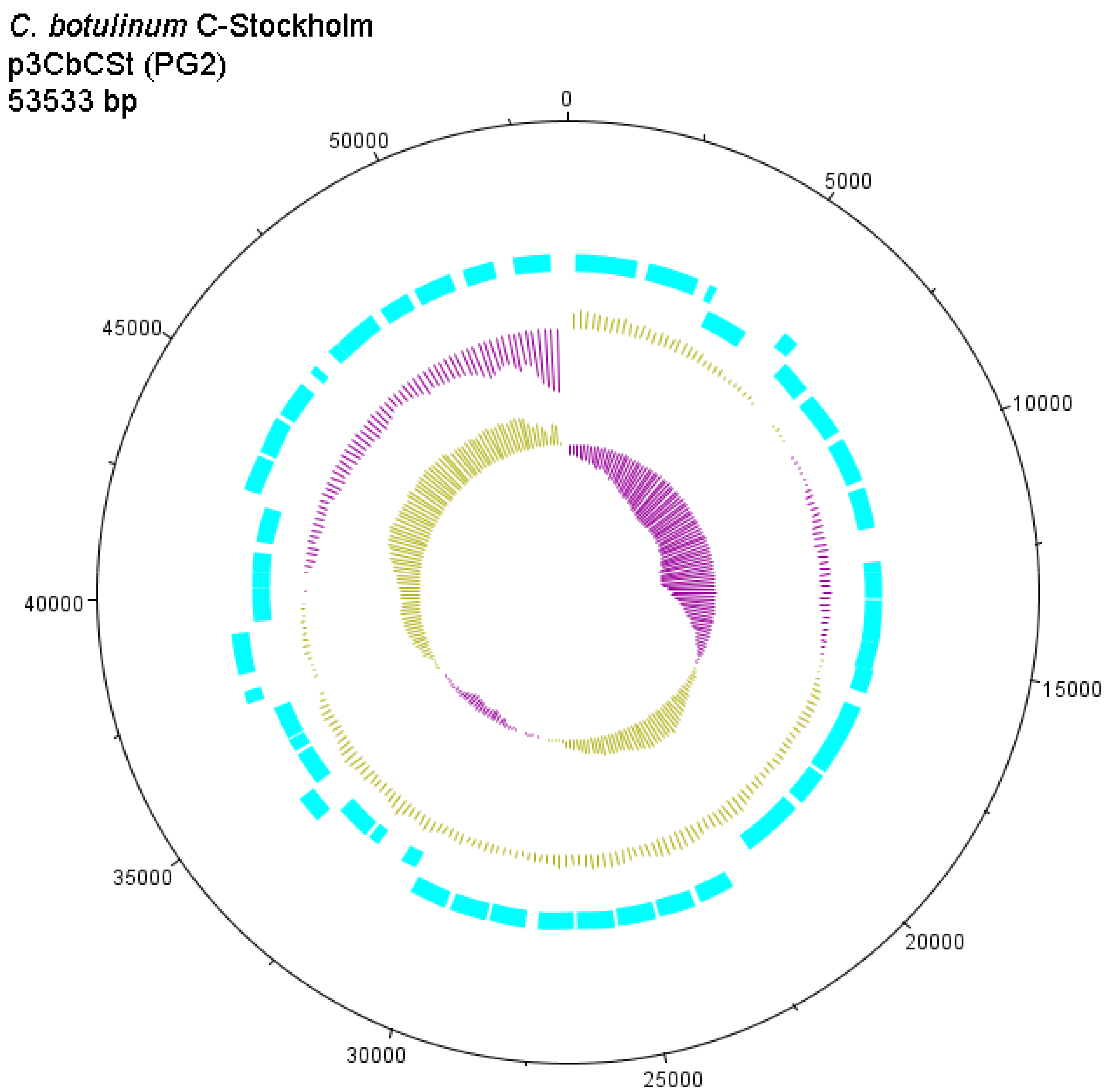
C. botulinum C-Stockholm
p3CbCSt (PG2)
53533 bp
0
5000
10000
15000
20000
25000
30000
35000
40000
45000
50000

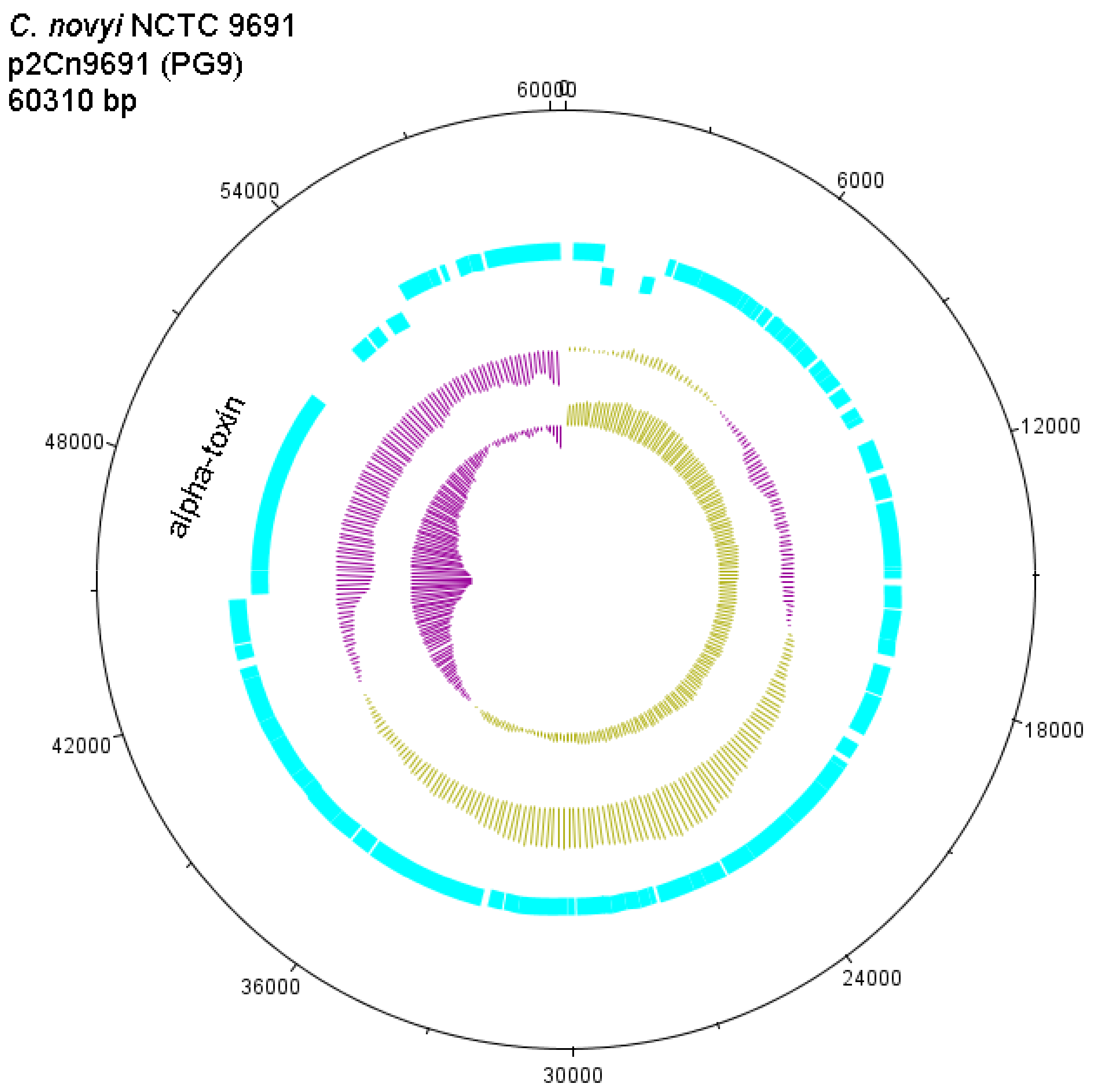
C. novyi NCTC 9691
p2Cn9691 (PG9)
60310 bp
60000
0
6000
12000
18000
24000
30000
36000
42000
48000
54000
alpha-toxin

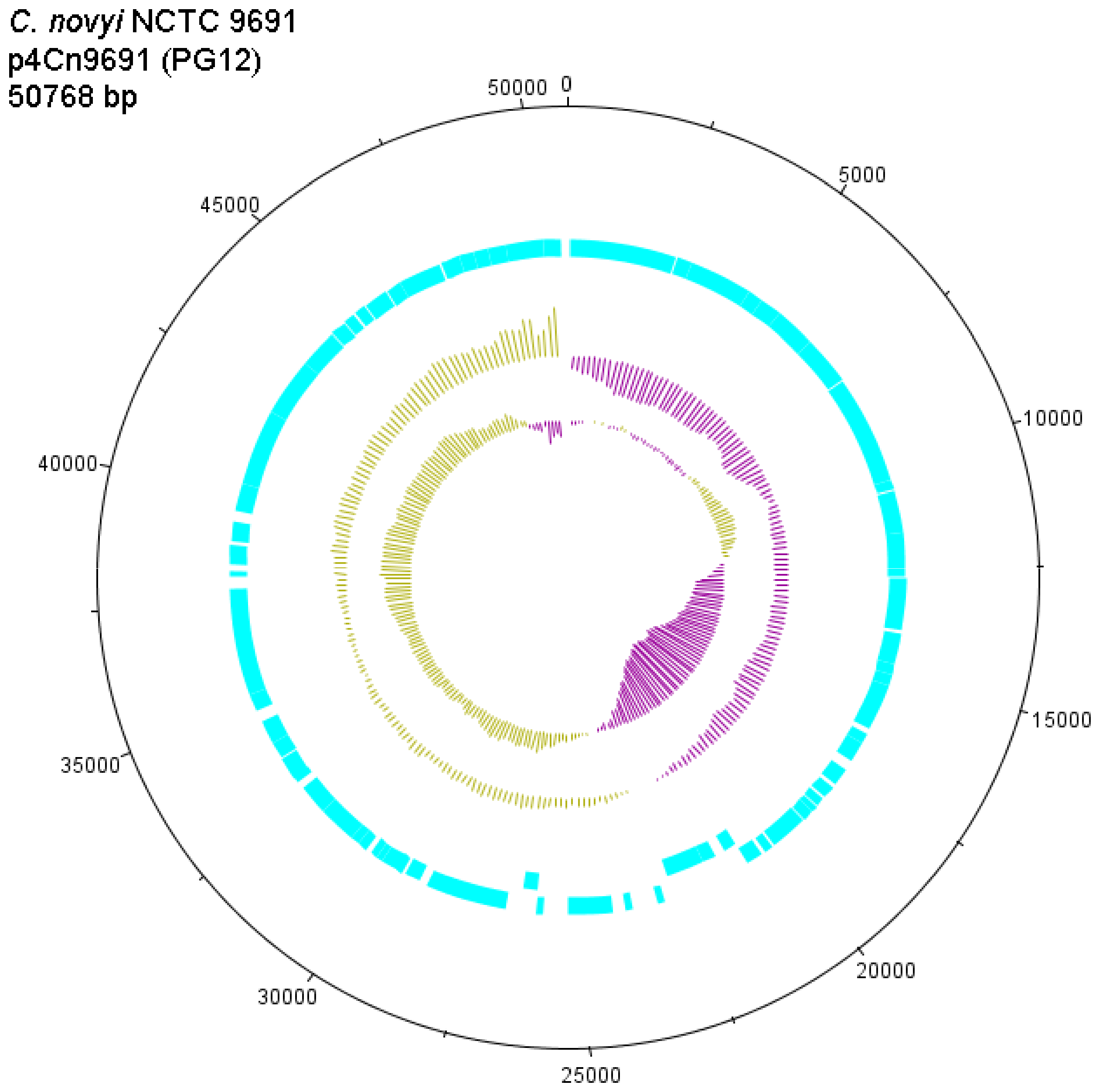

C. novyi NCTC 9691
p4Cn9691 (PG12)
50768 bp
0
5000
10000
15000
20000
25000
30000
35000
40000
45000
50000

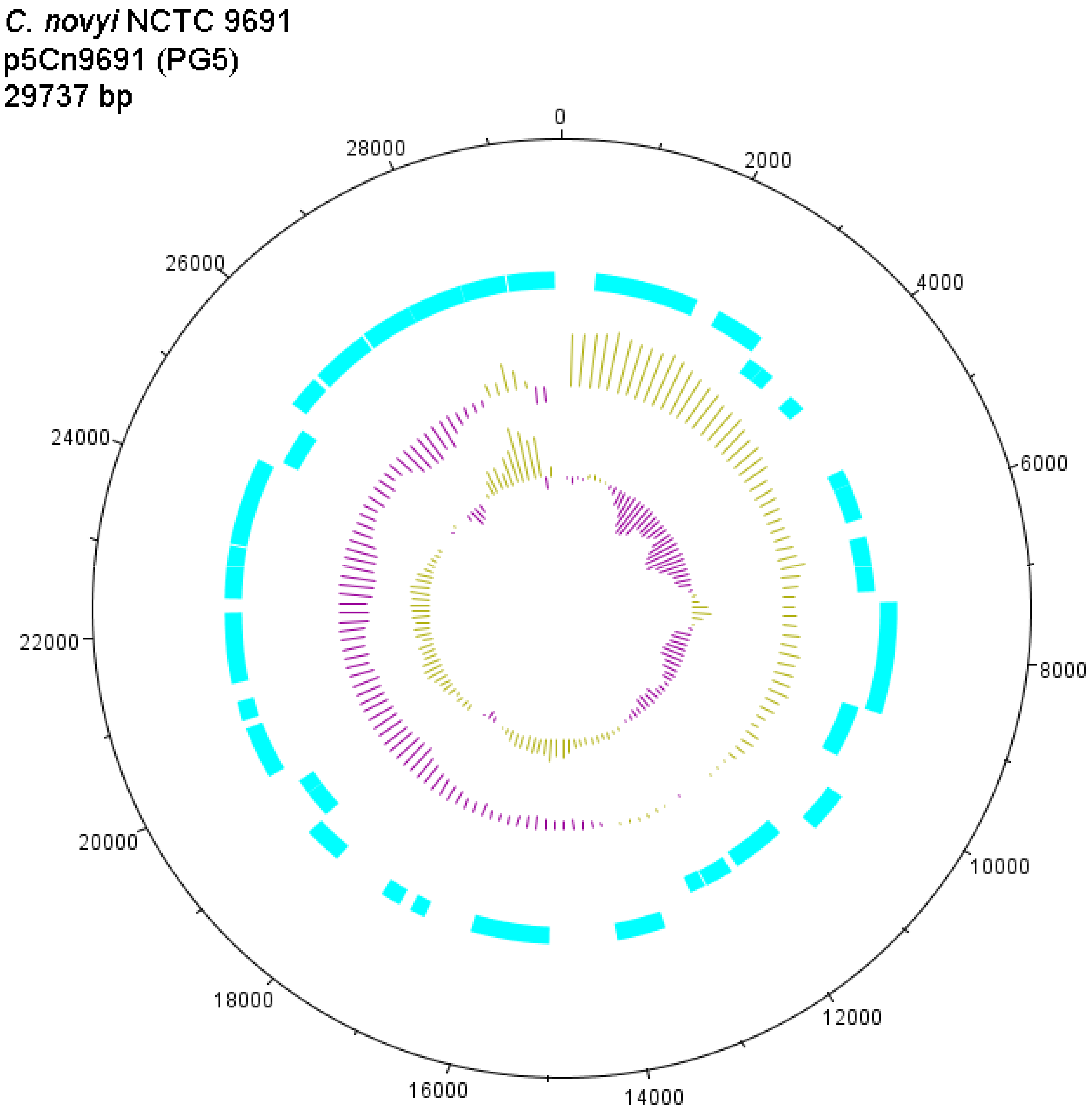
C. novyi NCTC 9691
p5Cn9691 (PG5)
29737 bp
0
2000
4000
6000
8000
10000
12000
14000
16000
18000
20000
22000
24000
26000
28000

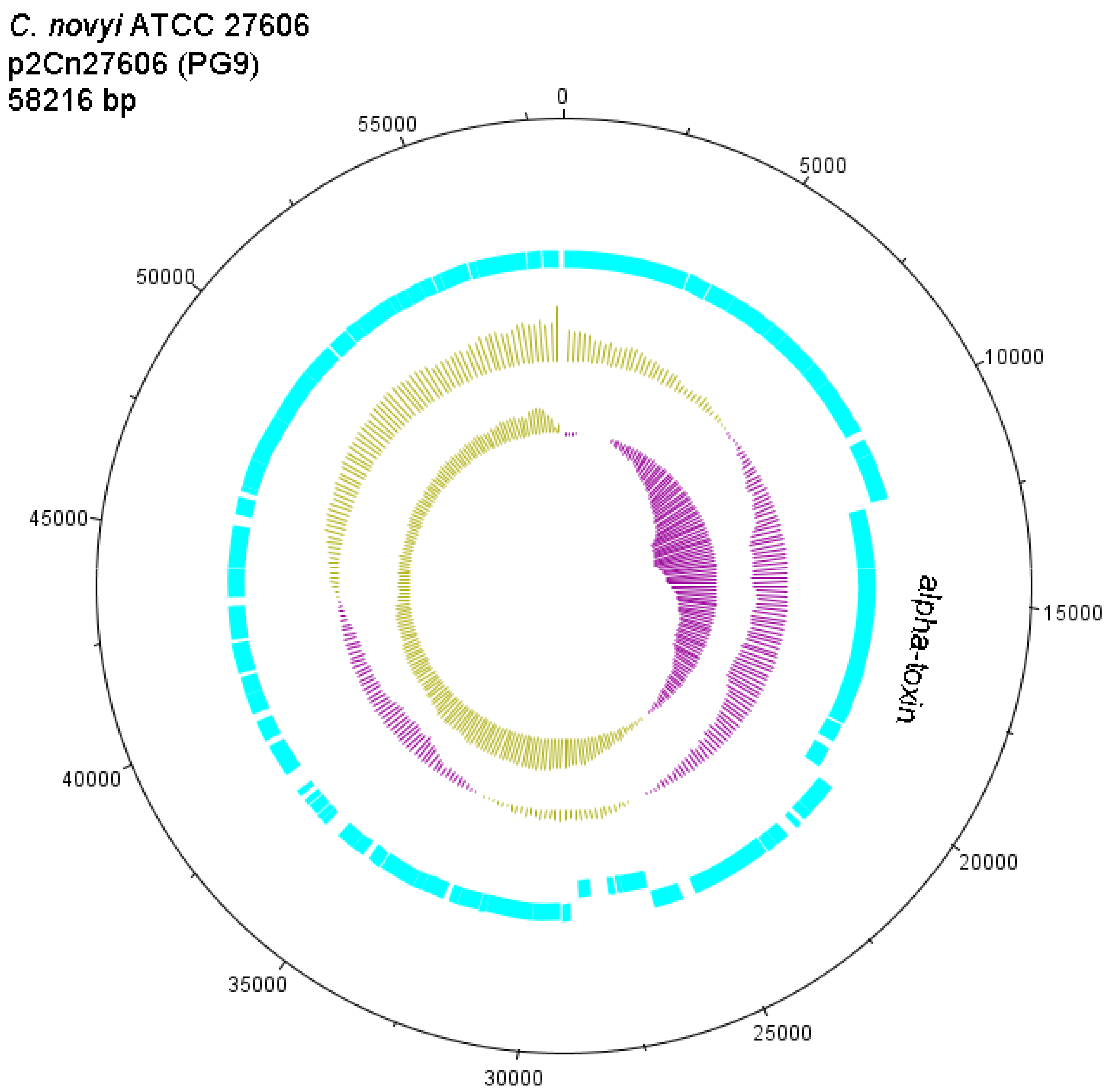

C. novyi ATCC 27606
p2Cn27606 (PG9)
58216 bp
0
5000
10000
15000
20000
25000
30000
35000
40000
45000
50000
55000
alpha-toxin

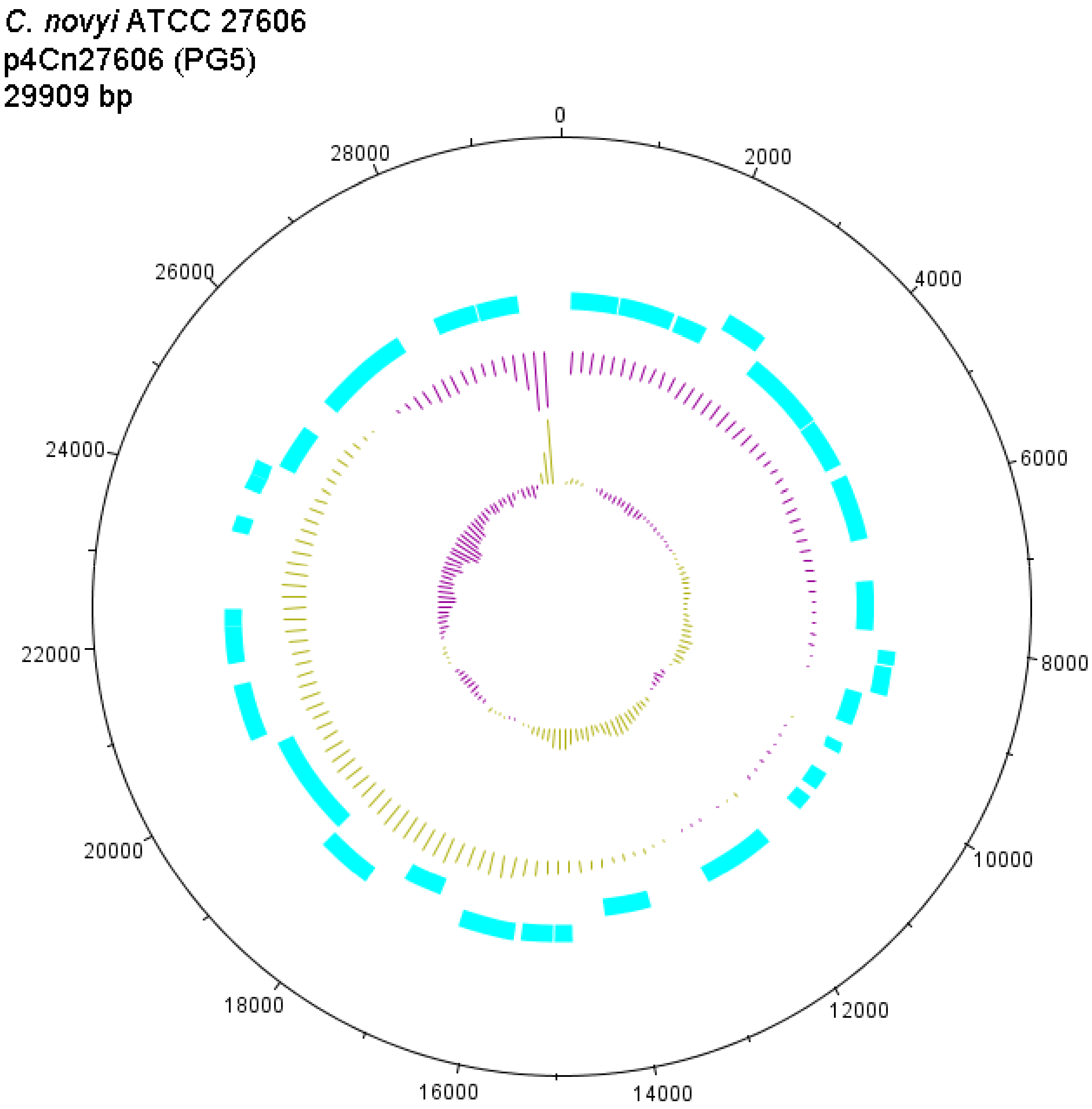

C. novyi ATCC 27606
p4Cn27606 (PG5)
29909 bp
0
2000
4000
6000
8000
10000
12000
14000
16000
18000
20000
22000
24000
26000
28000

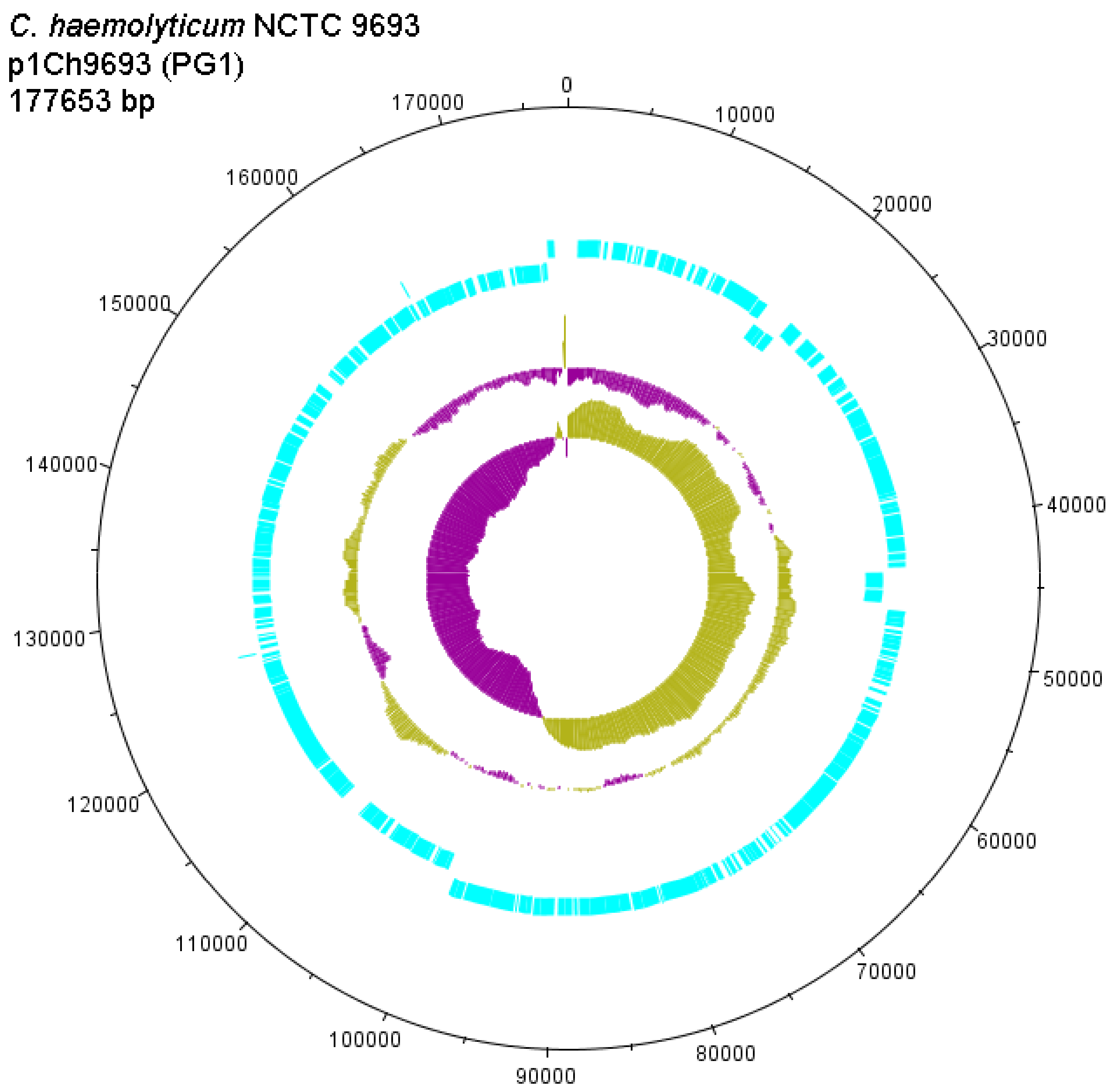
C. haemolyticum NCTC 9693
p1Ch9693 (PG1)
177653 bp
0
10000
20000
30000
40000
50000
60000
70000
80000
90000
100000
110000
120000
130000
140000
150000
160000
170000

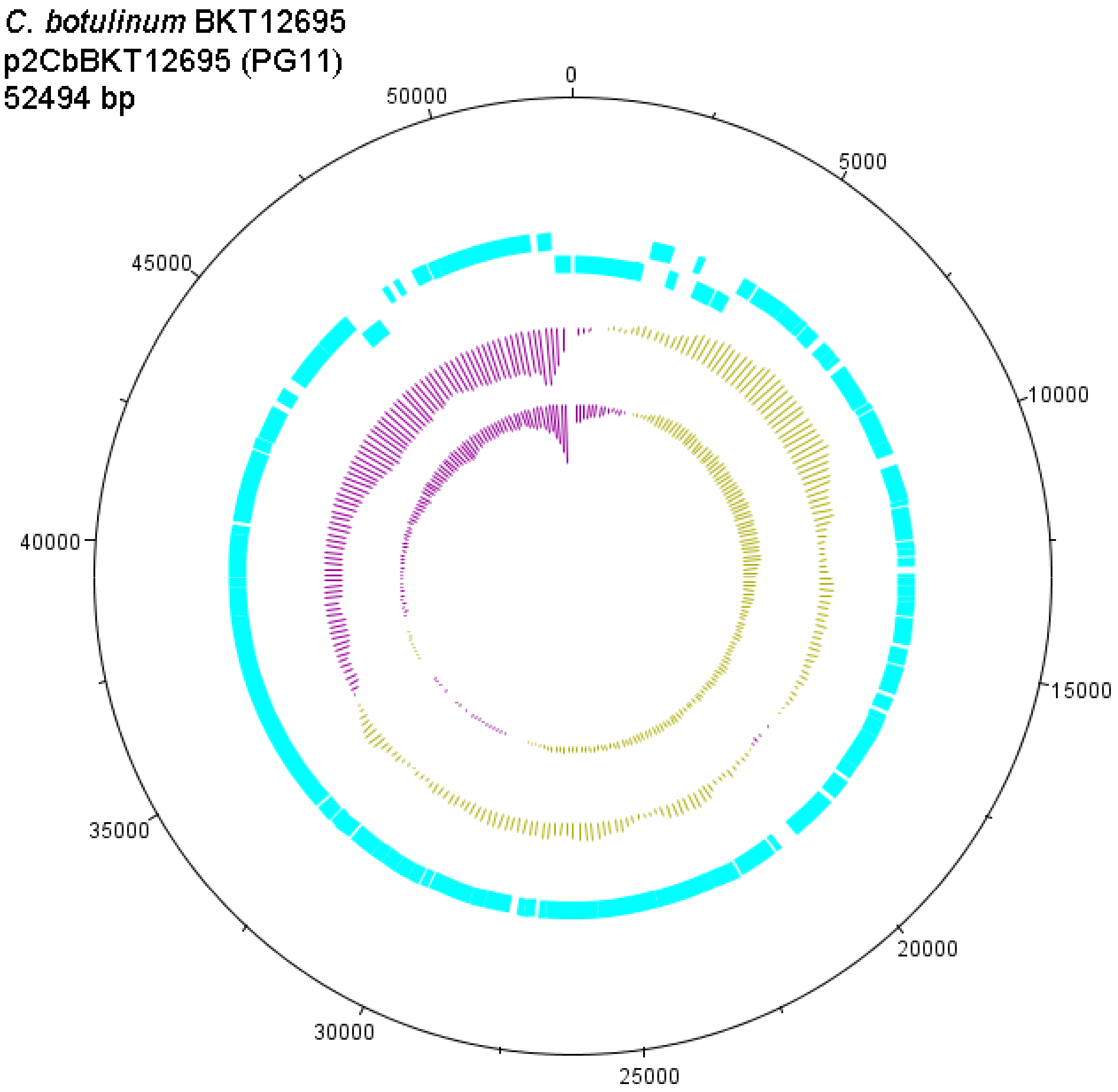

C. botulinum BKT12695
p2CbBKT12695 (PG11)
52494 bp
0
5000
10000
15000
20000
25000
30000
35000
40000
45000
50000

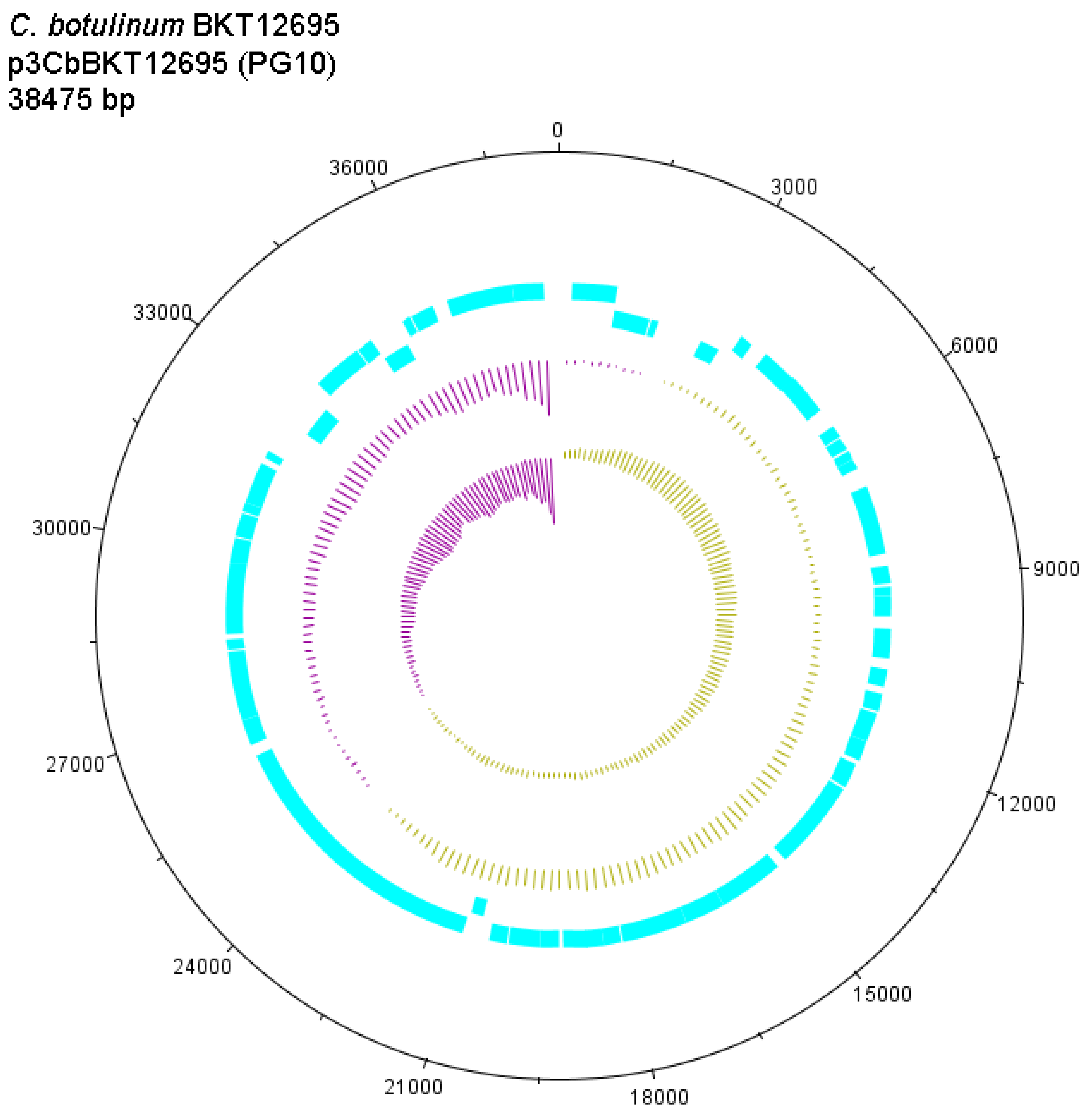

C. botulinum BKT12695
p3CbBKT12695 (PG10)
38475 bp
0
3000
6000
9000
12000
15000
18000
21000
24000
27000
30000
33000
36000

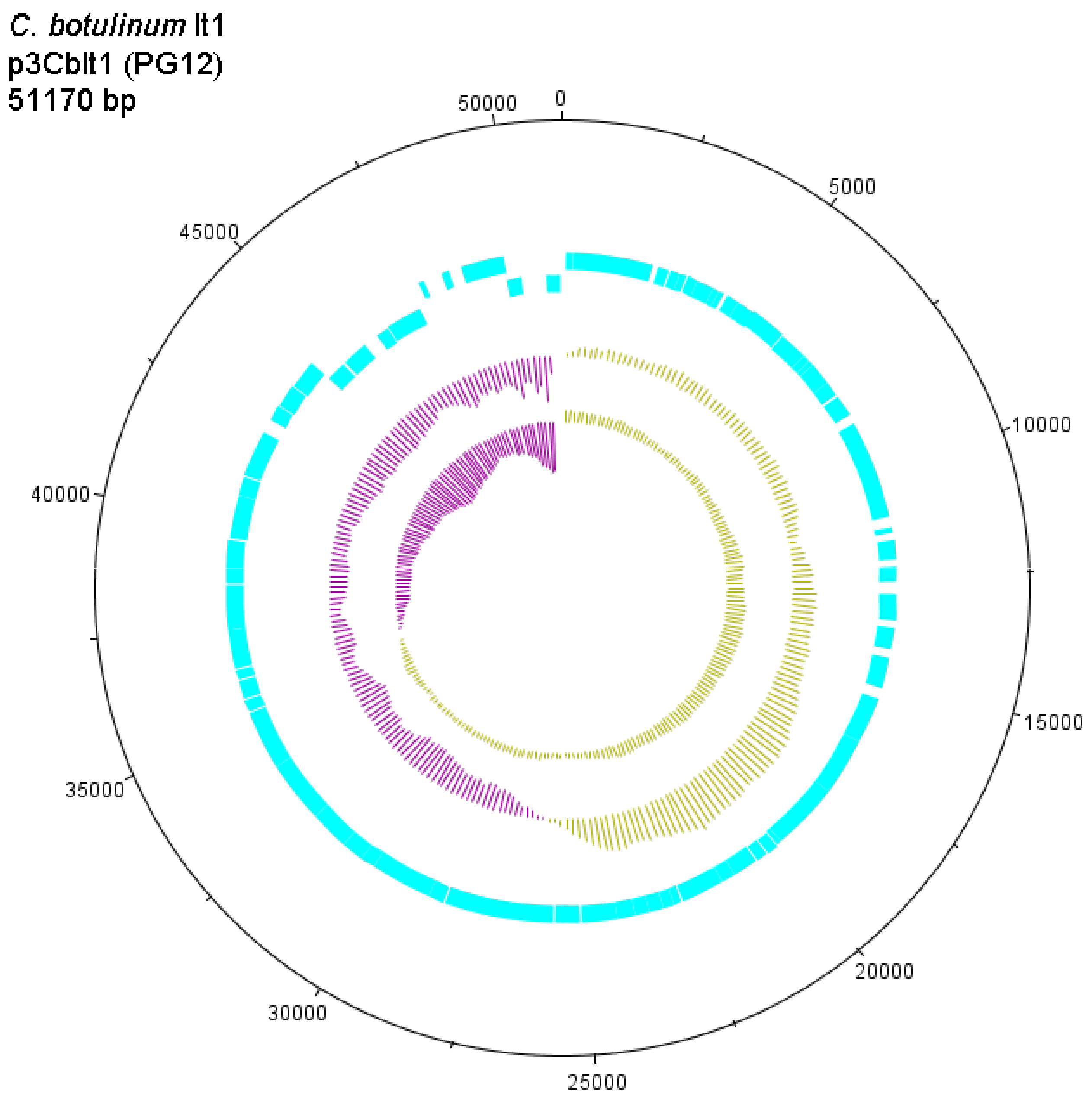

C. botulinum It1
p3CbIt1 (PG12)
51170 bp
0
50000
5000
45000
10000
40000
15000
35000
20000
30000
25000

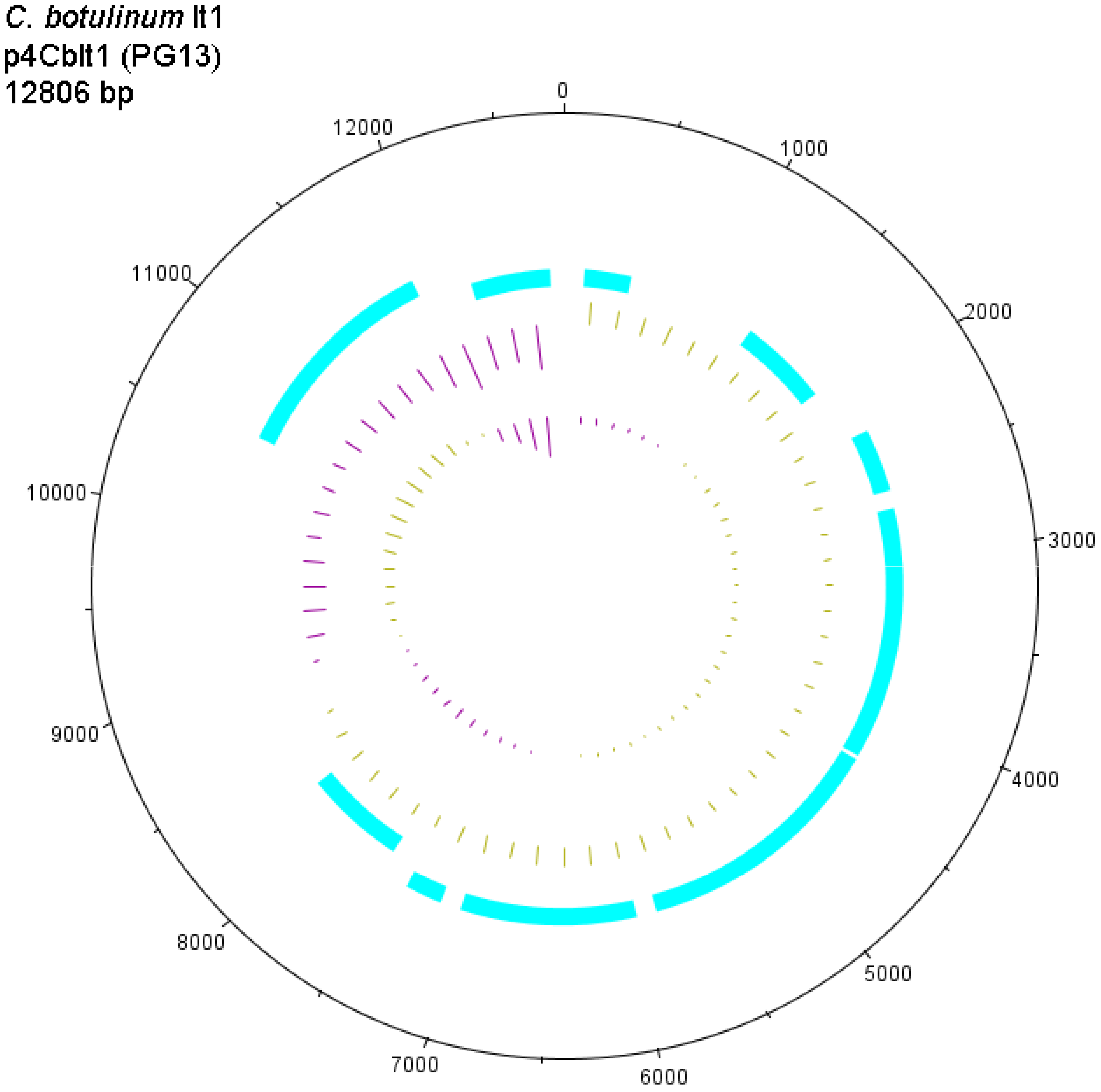

C. botulinum lt1
p4Cblt1 (PG13)
12806 bp
0
1000
2000
3000
4000
5000
6000
7000
8000
9000
10000
11000
12000

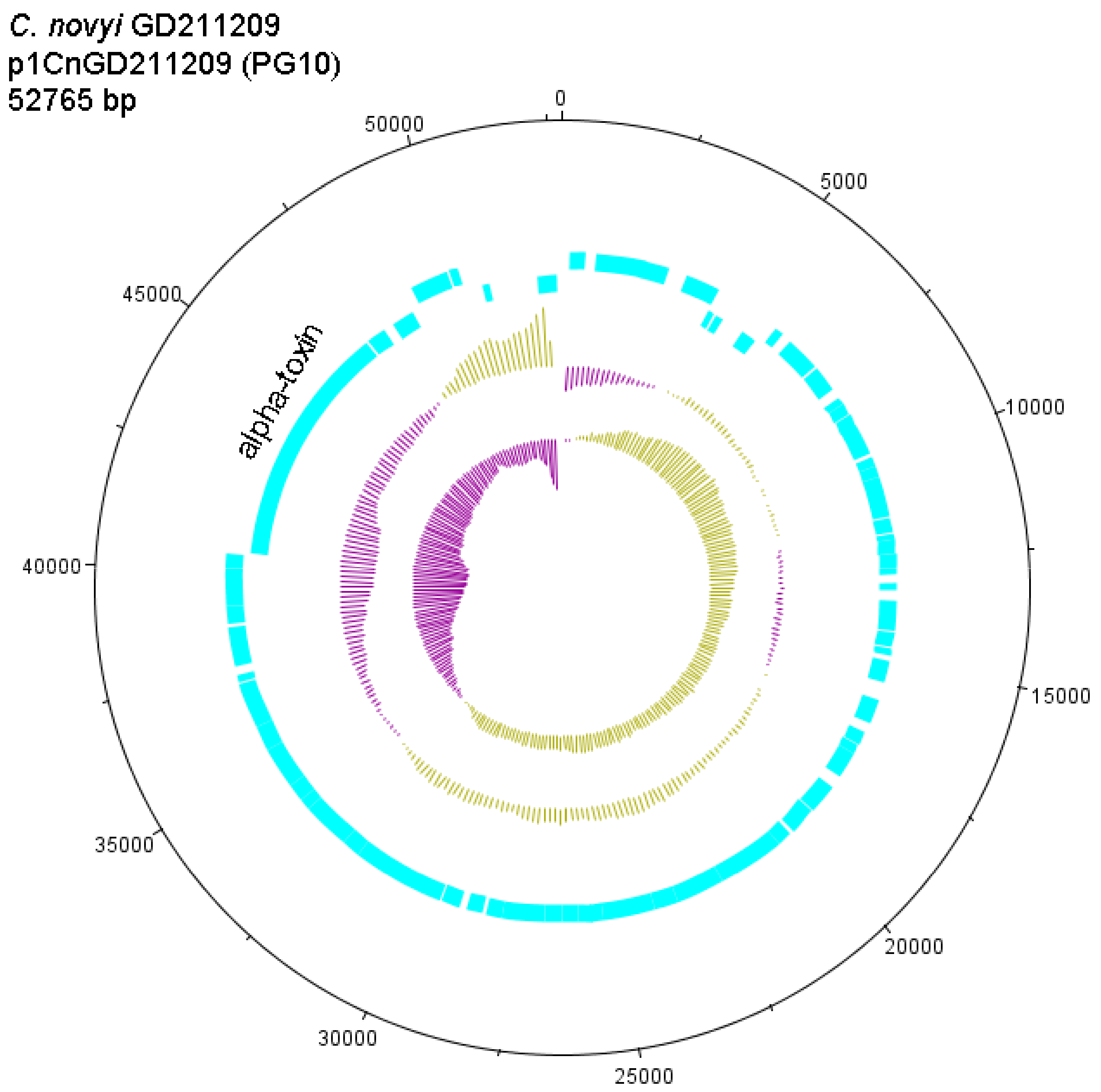

C. novyi GD211209
p1CnGD211209 (PG10)
52765 bp
0
5000
10000
15000
20000
25000
30000
35000
40000
45000
50000
alpha-toxin

C. novyi BKT29909
p1CnBKT29909 (PG10)
60360 bp

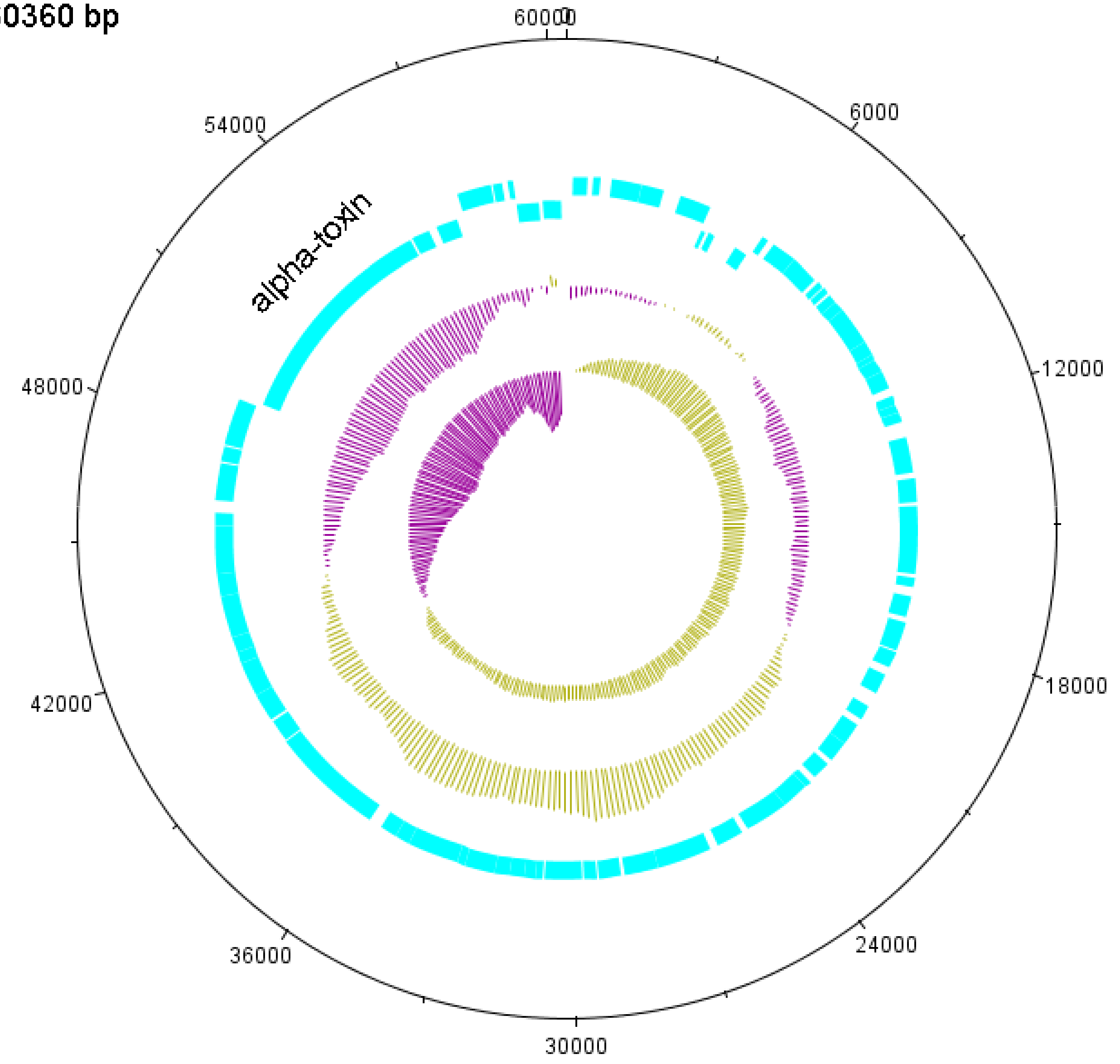

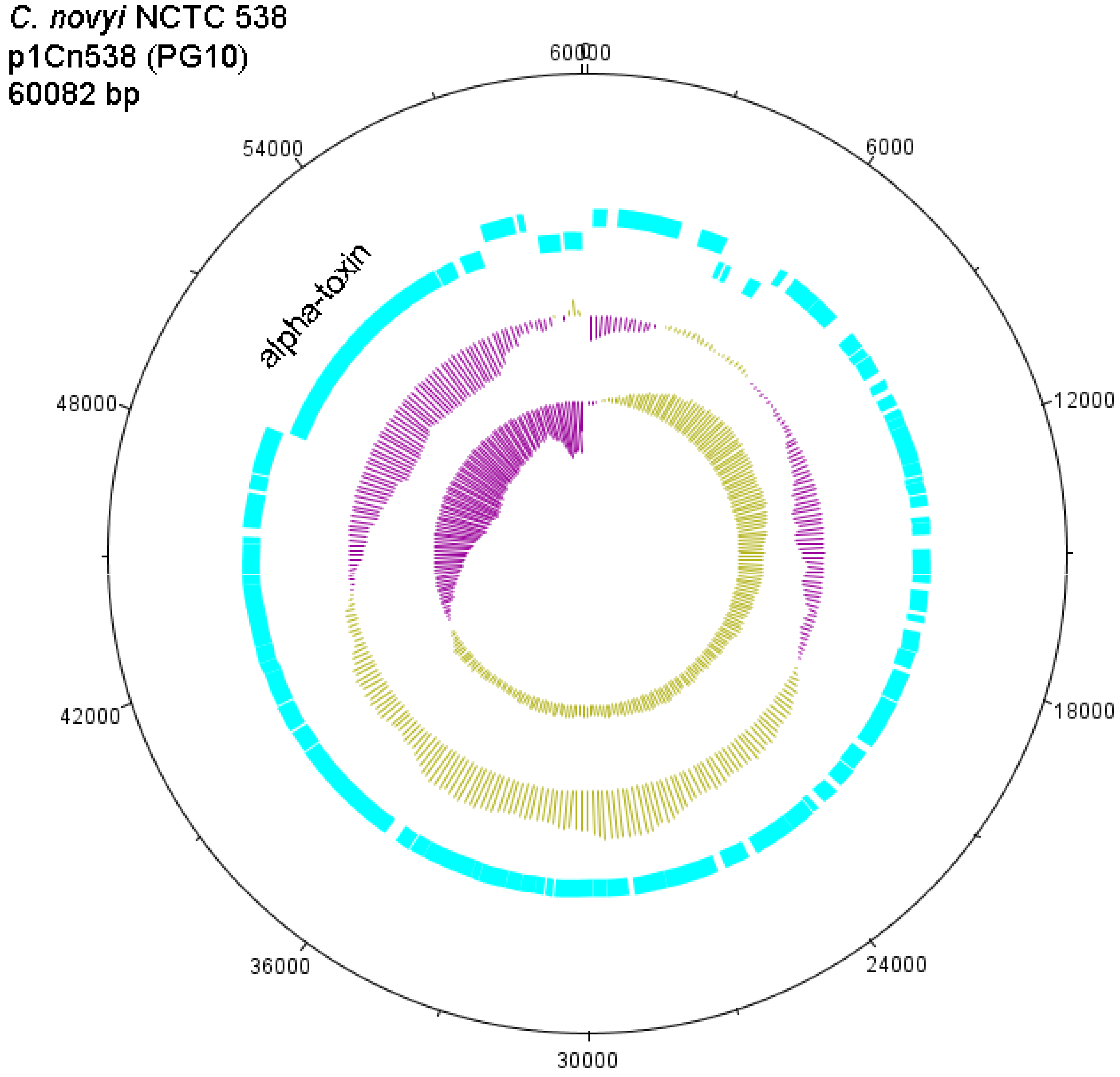

C. novyi NCTC 538
p1Cn538 (PG10)
60082 bp
60000
0
6000
12000
18000
24000
30000
36000
42000
48000
54000
alpha-toxin

C. novyi 4540
p1Cn4540 (PG10)
61041 bp

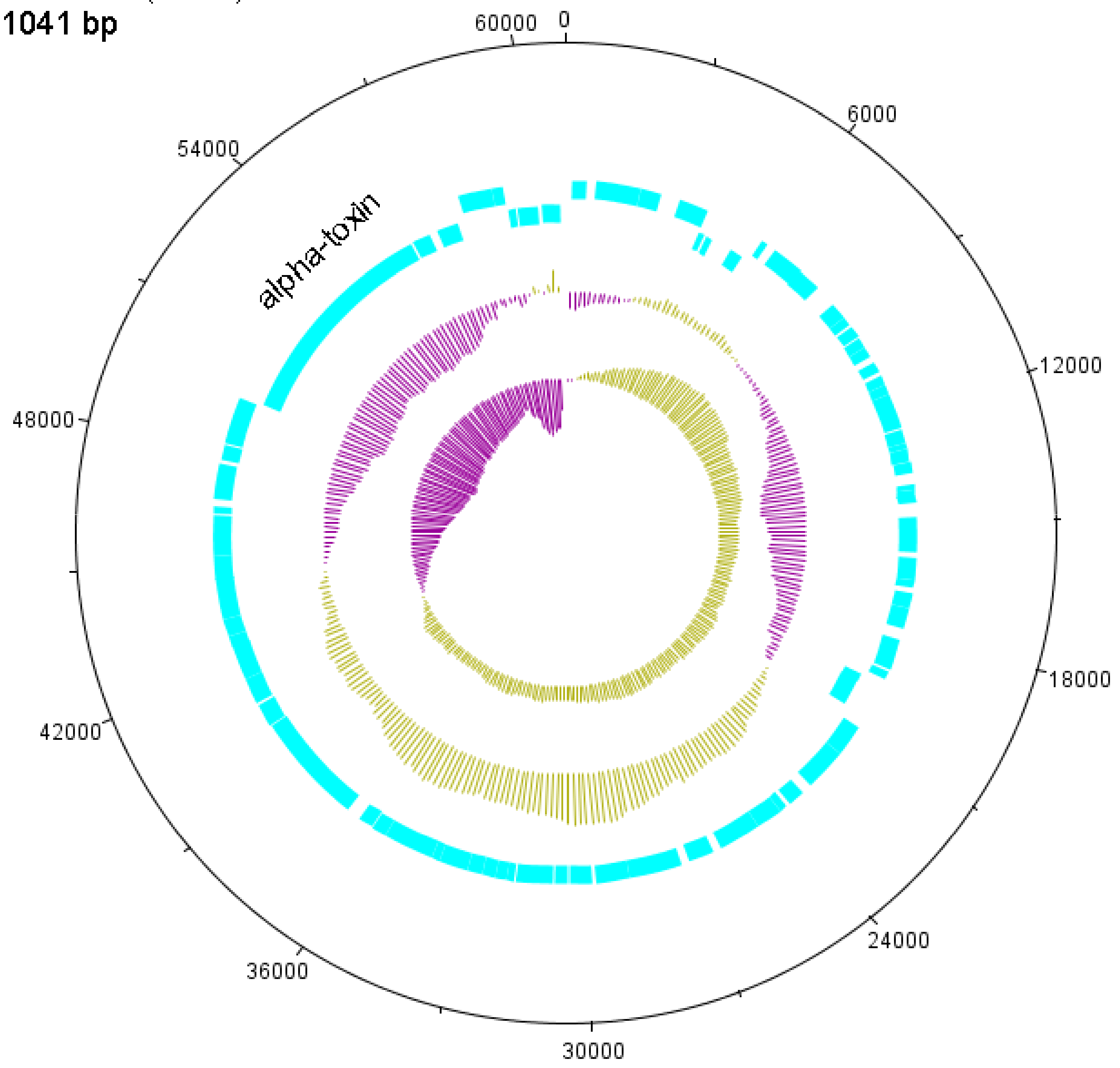

Supplement: Figure S1 — The completed plasmids. Circular representations of the plasmids completed in this study. The circle represents, beginning with the outermost, plus strand genes, minus strand genes, variation in GC and GC skew. (PDF) [file pone.0107777.s001.pdf]
