# Supplementary figures and images for: Plasmidome Interchange between Clostridium botulinum, Clostridium novyi and Clostridium haemolyticum Converts Strains of Independent Lineages into Distinctly Different Pathogens
Source: PLoS One. 2014 Sep 25;9(9):e107777. doi: 10.1371/journal.pone.0107777 (PMC4177856; doi:10.1371/journal.pone.0107777)

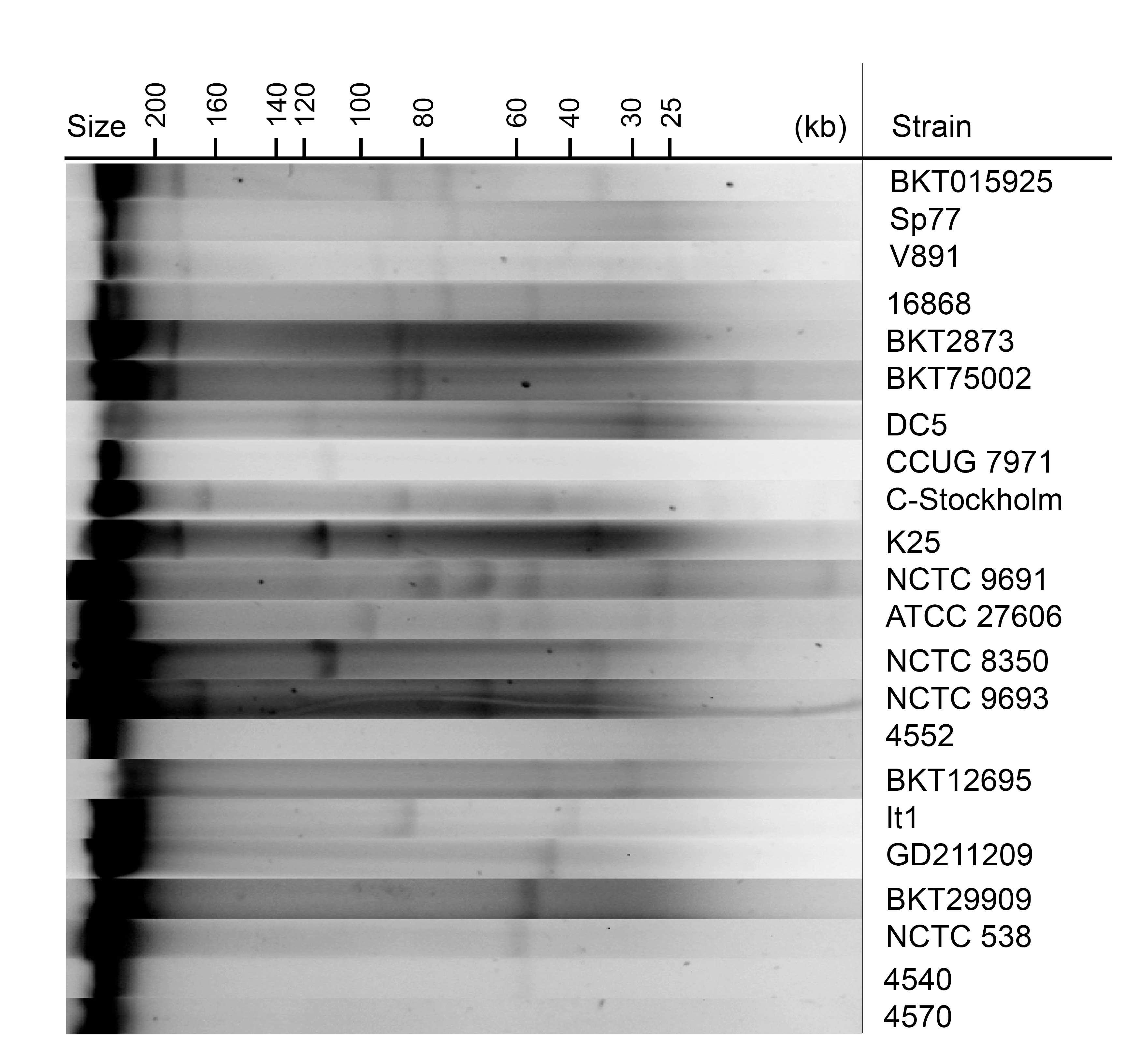

Supplement: Figure S2 — PFGE plasmid profiles. Plasmid separation by pulsed-field gel electrophoresis was used to estimate the number of plasmids and their sizes in each strain. DNA from strain BKT015925, where a completed genome was already available, was included as reference. Lambda Ladder PFG Marker was used as a standard and a size ladder was calculated using BioNumerics 6.5. (TIF) [file pone.0107777.s002.tif]

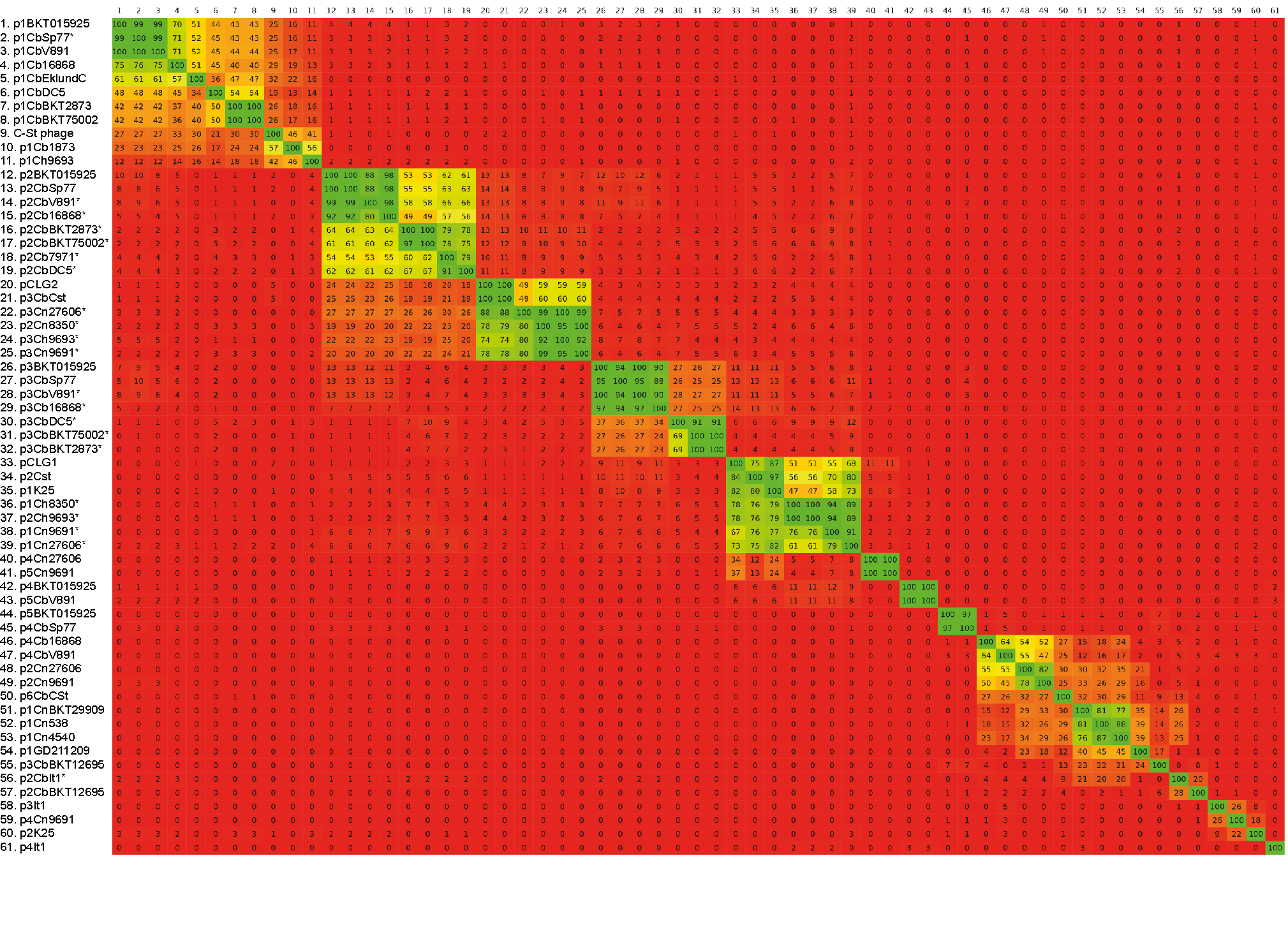

Supplement: Figure S3 — Similarity matrix showing the core sizes (40% normalized BLASTN score threshold) between C. novyi sensu lato plasmids. The full similarity matrix corresponding to the heat plot in Figure 2, showing the relative amount of shared genetic material between plasmids of C. novyi sensu lato. (TIF) [file pone.0107777.s003.tif]

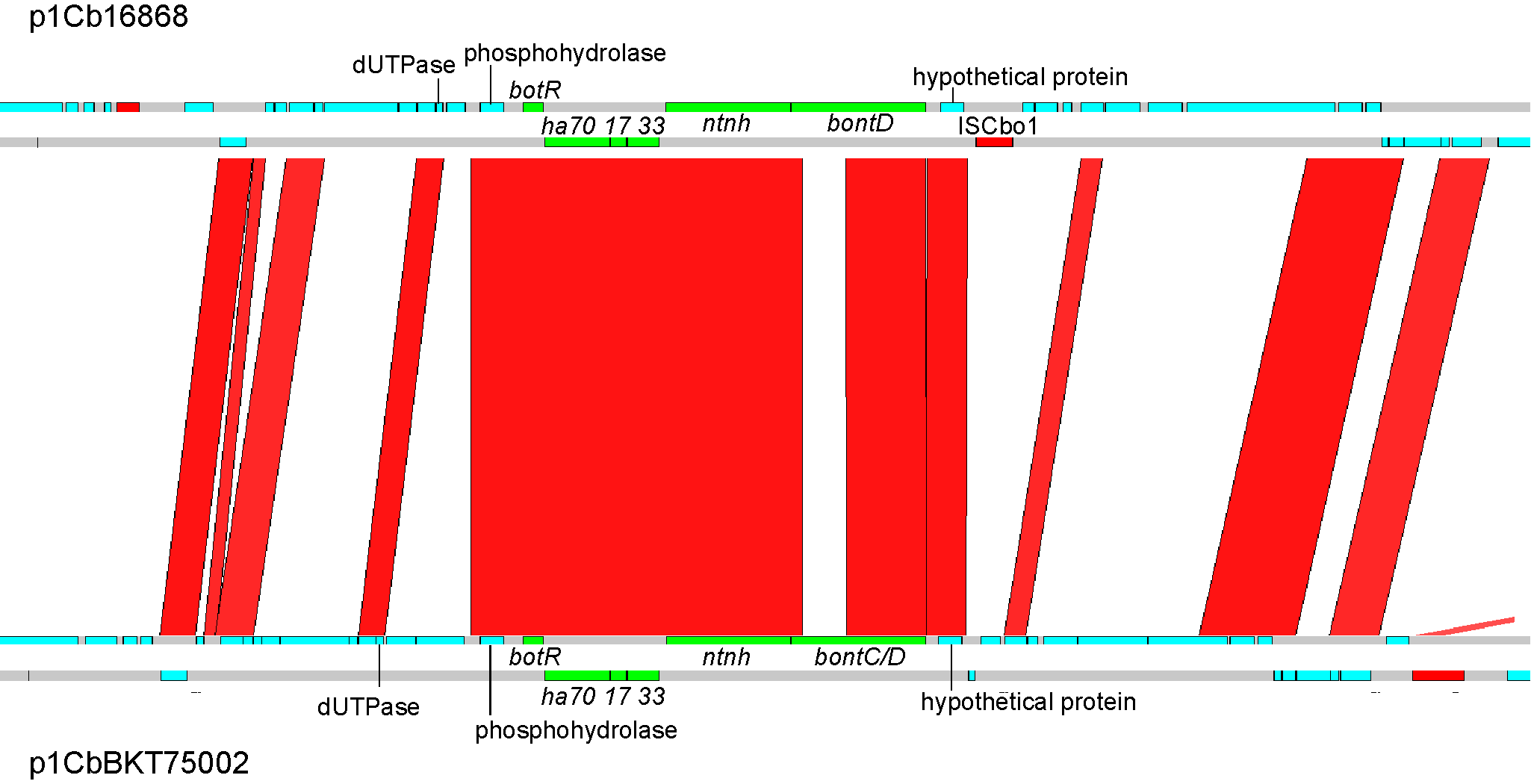

Supplement: Figure S4 — Alignment of the bont gene cluster and surrounding genes in two strains in lineage I. The aligned sequences are from type D strain 16868 and type C/D (subgroup Ia) strain BKT75002 (subgroup Ib). The bont cluster is marked in green and the complete IS element (ISCbo1) identified in strain 16868 is marked in red. (TIF) [file pone.0107777.s004.tif]
