# Supplementary material for: Plasmidome Interchange between Clostridium botulinum, Clostridium novyi and Clostridium haemolyticum Converts Strains of Independent Lineages into Distinctly Different Pathogens
Source: PLoS One. 2014 Sep 25;9(9):e107777. doi: 10.1371/journal.pone.0107777 (PMC4177856; doi:10.1371/journal.pone.0107777)
Supplement: Table S4 — The result of the plasmid curation experiment. (DOCX) [file pone.0107777.s008.docx]

**Table S4. The result of the plasmid curation experiment.**

The table shows which plasmid groups were removed by heat and novobiocin treatments. Plasmid groups PG3 and PG5 were the only two groups considered to be regular plasmids, which were cured by this treatment, whereas all the plasmid groups representing circular prophages were removed.

*not tested, but highly related genomes lack this plasmid, which indicates that it can be lost.

| Plasmid group | Strain analyzed | Plasmid | Lost in curation experiments |
| --- | --- | --- | --- |
| PG1 | BKT015925  NCTC 9693 | p1BKT015925  p1Ch9693 | Yes  Yes |
| PG2 | BKT015925  NCTC 9691  NCTC 9693  NCTC 8350  ATCC 27606 | p2BKT015925  p3Cn9691  p3Ch9693  p2Ch8350  p3Cn27606 | No  No  No  No  No |
| PG3 | BKT015925 | p3BKT015925 | Yes |
| PG4 | NCTC 9691  NCTC 9693  NCTC 8350  ATCC 27606 | p1Cn9691  p2Ch9693  p1Ch8350  p1Cn27606 | No  No  No  No |
| PG5 | NCTC 9691  ATCC 27606 | p5Cn9691  p4Cn27606 | Yes  Yes |
| PG6 | BKT015925 | p4BKT015925 | Yes |
| PG7 | BKT015925 | p5BKT015925 | Yes |
| PG8* |  |  |  |
| PG9 | NCTC 9691  ATCC 27606 | p2Cn9691  p2Cn27606 | Yes  Yes |
| PG10* |  |  |  |
| PG11* |  |  |  |
| PG12 | NCTC 9691 | p4Cn9691 | Yes |
| PG13* |  |  |  |

The table shows which plasmid groups that where discarded by heat and novobiocin treatments. Plasmid groups PG3 and PG5 were the only two groups considered to be ordinary plasmids, which were cured by this treatment, whereas all the plasmid groups representing circular prophages that were subjected to curation experiments were lost.

*not tested, but highly related genomes lack this plasmid, which indicates that they can be lost.
